# Honey: An Advanced Antimicrobial and Wound Healing Biomaterial for Tissue Engineering Applications

**DOI:** 10.3390/pharmaceutics14081663

**Published:** 2022-08-10

**Authors:** Joel Yupanqui Mieles, Cian Vyas, Enes Aslan, Gavin Humphreys, Carl Diver, Paulo Bartolo

**Affiliations:** 1 Department of Mechanical, Aerospace and Civil Engineering, The University of Manchester, George Begg Building, Manchester M1 3BB, UK; 2 Singapore Centre for 3D Printing, School of Mechanical and Aerospace Engineering, Nanyang Technological University, 50 Nanyang Avenue, Singapore 639798, Singapore; 3 Department of Machine and Metal Technologies, Gumusova Vocational School, Duzce University, Duzce 81850, Turkey; 4 Division of Pharmacy & Optometry, The University of Manchester, Oxford Rd., Manchester M13 9PL, UK; 5 Department of Engineering, Manchester Metropolitan University, All Saints Building, Manchester M15 6BH, UK

**Keywords:** honey, antimicrobial, hydrogen peroxide, antibiotic resistance, wound healing, tissue engineering

## Abstract

Honey was used in traditional medicine to treat wounds until the advent of modern medicine. The rising global antibiotic resistance has forced the development of novel therapies as alternatives to combat infections. Consequently, honey is experiencing a resurgence in evaluation for antimicrobial and wound healing applications. A range of both Gram-positive and Gram-negative bacteria, including antibiotic-resistant strains and biofilms, are inhibited by honey. Furthermore, susceptibility to antibiotics can be restored when used synergistically with honey. Honey’s antimicrobial activity also includes antifungal and antiviral properties, and in most varieties of honey, its activity is attributed to the enzymatic generation of hydrogen peroxide, a reactive oxygen species. Non-peroxide factors include low water activity, acidity, phenolic content, defensin-1, and methylglyoxal (*Leptospermum* honeys). Honey has also been widely explored as a tissue-regenerative agent. It can contribute to all stages of wound healing, and thus has been used in direct application and in dressings. The difficulty of the sustained delivery of honey’s active ingredients to the wound site has driven the development of tissue engineering approaches (e.g., electrospinning and hydrogels). This review presents the most in-depth and up-to-date comprehensive overview of honey’s antimicrobial and wound healing properties, commercial and medical uses, and its growing experimental use in tissue-engineered scaffolds.

## 1. Introduction

Honey has historically been used for medical purposes by various cultures since ancient times, due to its antimicrobial and regenerative properties. The ancient Egyptians utilised honey to treat wounds, which has been experimentally demonstrated to be efficacious in promoting healing and preventing infections [1]. For a long time, honey has been prescribed in traditional medicine such as the Indian Ayurvedic system for a variety of illnesses. Furthermore, there are direct references to honey consumption in the Bible (The Bible, Proverbs 24:13) and in the Quran (16:68–69), which mentions the “emerging drink from the bee’s bellies, in which there is healing for people”. The historic and continuing global usage of honey as a therapeutic agent pertains to its remarkable antimicrobial efficacy and tissue-regenerative properties.

Although used traditionally in wound treatments and other illnesses, the advent of modern medicine and antibiotics has reduced its medical usage. However, the widespread use of antibiotics has led to a significant rise in antibiotic-resistant infections globally, which by 2050 could lead to 10 million deaths per year if new treatments are not developed [2,3,4]. Subsequently, the discovery and development of new antibiotics is a global priority. This has initiated a re-evaluation of the clinical use of honey in conjunction with a growing awareness and understanding of the material properties, composition, and mechanisms of the antimicrobial action of honey.

Honey is produced by eight species of bee within the genus *Apis*, which represents a small fraction of the approximately 15,000 species of bee. However, the world population of western honeybee (*Apis mellifera*), widespread across the world, is decreasing due to several factors, including, but not limited to: climate change, the use of pesticides in agriculture, disruptions to their specialised gut microbiome, and the prevalence of the Deformed Wing Virus associated with the ectoparasitic *Varroa destructor* mite [5,6,7,8,9,10].

Honeybees produce honey through a complex process beginning with the collection of floral nectar (floral or blossom honey) or sugar-rich secretions from insects (honeydew honey) as raw materials. These are stored and processed in their hives. The bees dehydrate, add their own compounds to, and modify the nectar through the secretion of specific enzymes to break down sugars. The modified nectar matures and develops into honey. Honey is a viscous and concentrated aqueous sugar solution generally comprising fructose (~40%), glucose (~30%), sucrose (~5%), small quantities of disaccharides (e.g., maltose, isomaltose, and turanose), and water (~20%). It is worth noting that these percentages are only representative and can substantially differ due to botanical sources, nectars, and seasons [11]. In addition, a variety of proteins, amino acids, minerals, enzymes (e.g., glucose oxidase and invertase), vitamins, and polyphenols are also present [12,13,14]. The composition and properties of honey depend on the surrounding environment of the hive and the metabolic activity of the bees. For example, the collection of nectar can either be predominately monofloral (single species of plant) or multifloral (multiple species of plant) which can give rise to unique properties and distinctive tastes.

The bactericidal efficacy of honey was reported more than a century ago by Van Ketel [15], and those findings prompted extensive research on honey over the next decades. The effectiveness of honey’s antimicrobial activity varies greatly depending on its geographical and botanical source, and its harvesting, processing, and storage conditions. Similarly, the source also determines the specific biochemical factors that provide honey with antimicrobial properties, and consequently the clinical effectiveness on different microbial strains.

The predominant antimicrobial activity of the majority of honeys can be attributed to the generation of hydrogen peroxide (H_2_O_2_) [16,17,18,19,20,21,22]. The presence of the enzyme, glucose oxidase (GOx), is fundamental to produce H_2_O_2_ and is secreted into the nectar by bees during the preparation of honey. The enzymatic oxidation of glucose via GOx generates gluconic acid and H_2_O_2_ species [20,22,23,24,25,26]. The enzyme presents no activity in raw honey, due to a lack of free water, to initiate the peroxide-dependent antimicrobial mechanism the honey needs to be diluted. Other important antimicrobial features responsible for the non-peroxide activity of honey include low water content (osmotic effect), low pH (acidic environment), phenolic compounds, bee defensin-1 (Def-1), and methylglyoxal (MGO) (in *Leptospermum*-derived manuka honey).

Honey is mainly used as a topical application on wounds where the antibacterial properties of honey are essential. The high viscosity of honey provides an effective hydrated barrier between the wound site and external environment. A variety of wound types have been treated with honey, such as burns, trauma, and chronic wounds [27,28,29]. However, the wound healing process is a complex multi-factorial cascade of events that if interrupted by infection or specific disease states (e.g., diabetes) can lead to the development of chronic wounds, recurrent infections, amputation/limb salvage, and life-threatening conditions. Growing antibiotic resistance further complicates the problem and can lead to preventable deaths from the infection of wound sites and sepsis. Subsequently, there is a critical need for new treatment options. Natural products such as honey can be part of the solution and is a promising candidate to create novel antimicrobial wound dressings.

Honey has been used in combination with traditional wound dressings but presents some limitations, such as being absorbed by the dressing, poor penetration into the wound site, and short-term antimicrobial action. The manufacturers of impregnated dressings are striving to improve their delivery mechanism. However, the limitations of traditional delivery methods of honey to the wound site have highlighted the need for new innovative routes of delivery, with methodologies such as electrospun fibres and hydrogels actively being explored [29,30,31,32,33]. This can enable the honey to remain in direct contact with the wound bed and provide a persistent and long-term release of antimicrobial agents. Furthermore, the presence of reactive oxygen species (ROS) such as H_2_O_2_ has been shown to promote wound healing by encouraging cellular repair processes and tissue regeneration [20,34]. Thus, the use of honey, honey-derived, and honey-inspired products in tissue engineering applications combined with other biomaterials may enable its use in a variety of other clinical situations outside wound care, where the combination of antimicrobial properties and tissue regeneration is desirable.

This review highlights the main antimicrobial characteristics and mechanisms of action of honey, including peroxide and non-peroxide processes, and its effectiveness against bacterial, fungal, and viral pathogens. The role of honey in wound healing and tissue regeneration is discussed. Finally, key challenges associated with the clinical implementation of honey and potential future directions for honey-based and -inspired materials are considered.

## 2. Antimicrobial Properties

**Figure 1:**
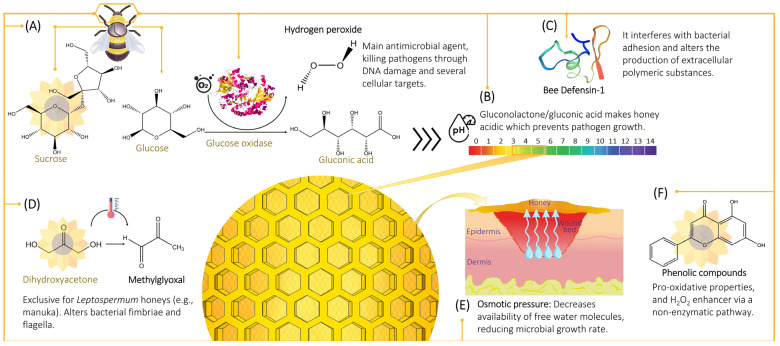
Key antimicrobial components of honey. (**A**) Sucrose from flowers is broken down by the bee into glucose and fructose. The bee’s hypopharyngeal glands secrete GOx. Glucose is then oxidised by the oxidised form of GOx, which results in the production of gluconolactone/gluconic acid and H_2_O_2_. Most of honey’s antimicrobial activity comes from H_2_O_2_, killing pathogens through DNA damage and several cellular targets. (**B**) Honey is acidic with an average pH of 3.91 (ranges between 3.4 to 6.1), which makes it powerful against microbial strains with an optimum pH of growth around 7. Acidity predominantly arises from gluconolactone/gluconic acid. (**C**) Bee Def-1 is an antibacterial peptide originating in the bee’s hypopharyngeal gland. It acts by interfering with bacterial adhesion to a surface, or in the early biofilm stage by inhibiting the growth of attached cells; and by altering the production of extracellular polymeric substances. (**D**) MGO is generated in honey during storage by the non-enzymatic conversion of dihydroxyacetone, a saccharide found in high concentrations in the nectar of *Leptospermum* flowers. The antimicrobial activity of MGO is attributed to alterations in bacterial fimbriae and flagella, which obstruct the bacterium’s adherence and motility. (**E**) Honey is a super-saturated solution of sugars. The strong interaction between these sugars with water molecules prevents the abundance of free water molecules (low water activity) available for microbes to grow. (**F**) The combination of different phenols act as an enhancer of honey’s antimicrobial efficacy. In alkaline conditions (pH 7.0–8.0), polyphenols can display pro-oxidative properties, inhibiting microbial growth by accelerating hydroxyl radical formation and oxidative strand breakage in DNA. They could also support the production of considerable amounts of H_2_O_2_ via a non-enzymatic pathway.

**Figure 2:**
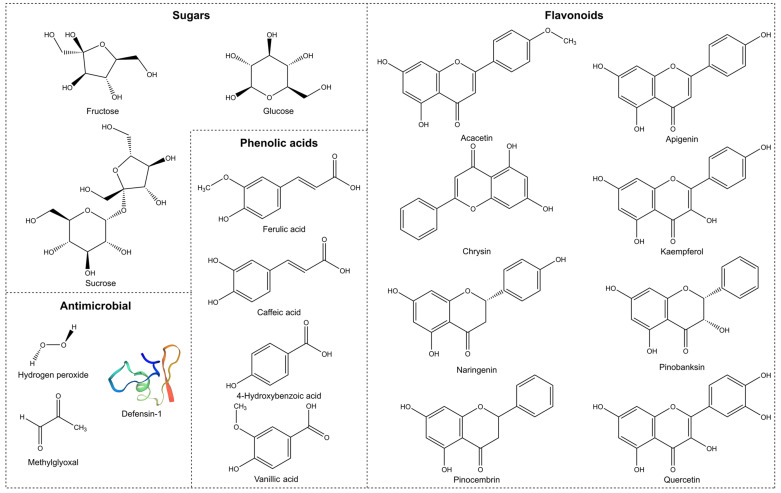
Compounds that can contribute to the overall antimicrobial properties of honey, including H_2_O_2_, def-1 (Swissmodel, P17722) [35], MGO (*Leptospermum* honeys only), flavonoids, phenolic acids, and sugars.

The antimicrobial activity of honey is multi-factorial but has historically been poorly understood. However, within the past century, honey’s antimicrobial properties have been identified and can be broadly attributed to peroxide and non-peroxide activities (Figure 1), with a range of compounds contributing to these activities (Figure 2).

### 2.1. Hydrogen Peroxide

Dold et al. [36] proposed the first detailed assay for the detection of antibacterial action in natural materials including honey. They claimed that ‘inhibine’ was honey’s antibiotic principle, which was subsequently validated by Prica [37] and Plachy [38] through different assays with bacterial filters, such as Seitz filters and dialysers. Later, a study using *Staphylococcus aureus* and nutrient Agar plates also attributed the inhibition effect to inhibine, which remained an unknown factor [39]. The first investigations on the inhibine activity had already discarded sugars, acids, nitrogen compounds, enzymes, vitamins or other known constitutes of honey as the main factors responsible for its antimicrobial activity [38].

Adock [40], motivated by the finding that honey’s antibiotic effect is strongly affected by heat and light, similar to H_2_O_2_, suggested the investigation of H_2_O_2_ as the main antimicrobial agent. Subsequently, White et al. [41] claimed that H_2_O_2_ was the inhibine previously described by Dold et al. [36]. This claim was supported as the experimental data on inhibine were consistent with H_2_O_2_, which was found to be produced by GOx. Furthermore, spectrophotometric assays demonstrated that the H_2_O_2_ production was directly proportional to the enzymatic activity, and most notably, to the so-called ‘inhibine number’, the semi-quantitative factor that was used to indicate the degree of antibacterial activity of a sample. Furthermore, only unheated samples showed H_2_O_2_ presence, confirming previous claims that heat inhibited the inhibine activity.

The role of H_2_O_2_ in the antimicrobial activity of diluted honey became more evident when experiments showed that the removal of all or most of the antimicrobial activity was achieved by the addition of enzymes (e.g., catalase) that decompose H_2_O_2_ [40,42]. Similarly, enzymatic activity and H_2_O_2_ release satisfied previous research works performed with bacterial filters when it was believed honey’s antimicrobial agent was ‘inhibine’ [17].

Dustmann [43] further demonstrated the direct relationship between H_2_O_2_ concentration and the inhibitory action in dialysed honey solutions. This study also highlighted the relevance of the hypopharyngeal glands of bees, which were found to secrete glucose oxidase.

#### 2.1.1. Hydrogen Peroxide Production

GOx, systematic name β-D-glucose: oxygen 1-oxidoreductase and EC number 1.1.3.4, is part of the oxidoreductase group of enzymes within the sub-class of dehydrogenases [44,45]. These enzymes catalyse the removal of hydrogen atoms from substrates (donors) to acceptors, and in the process, acceptors are reduced, and donors are oxidised. Glucose oxidase is specifically classed as an oxidase enzyme, as oxidase reactions exclusively consist of molecular oxygen as a hydrogen acceptor.

**Figure 3:**
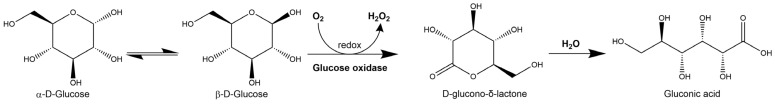
Schematic representation of the enzymatic reaction between glucose oxidase and glucose to produce H_2_O_2_ and gluconic acid.

The oxidised form of GOx catalyses the extraction of two hydrogen atoms from glucose’s -CHOH group, forming reduced glucose oxidase and gluconolactone (Figure 3). Then, the gluconolactone is hydrolysed to gluconic acid and the reduced enzyme is re-oxidised by molecular oxygen [44]. This reaction results in the formation of gluconic acid and hydrogen peroxide [45].

GOx is present in the hypopharyngeal glands of the honey bee, and its interaction with the glucose solution makes it become acidic shortly after it leaves the body of the bee [46]. When Schepartz and Subers [47] isolated GOx from honey, it was found that this enzyme had similar properties to the enzyme found in the bee glands, concluding that it was secreted into the nectar during the production of the honey [48].

Important studies revealed that GOx showed little or no activity in full-strength honey, releasing hydrogen peroxide only when it is diluted [41,49]. Dilution leads to an increase of 2500–50,000 times in the amount of H_2_O_2_. This finding contradicted claims that H_2_O_2_ was not responsible for the antimicrobial activity, based on the low concentration of H_2_O_2_ present in the undiluted honey [17]. Possible explanations for the limited activity of GOx may be the minimum amount of free water, as well as honey’s unfavourable pH. GOx presents optimum activity at pH of 6.1, good activity between 5.5–8 pH, then rapidly decreasing with pH below 5.5, and being inactive at pH 4 [47]. Gauhe [46] claimed that the acid generated by GOx due to its interaction with the glucose solution is gluconic acid. This is where most of honey’s acidity comes from, but it appears that the inhibition of enzyme activity arises from the resulting pH due to other compounds in honey, rather than the reaction with the gluconic acid itself.

Subers and co-authors [47,49] demonstrated honey’s GOx dependency on glucose concentration to become active and identified that a concentration of approximately 1.5 M obtained an optimum activity of GOx. This requirement is easily met by ripened honeys, as they usually have glucose concentrations of about 2 M. However, ripened honey should not be used directly as antibiotic treatment. For adequate antimicrobial activity, high substrate dilutions should be utilised as they relate better to body fluids than honey diluted to low levels. The effectiveness of a honey type for antibiotic treatment should be assessed by its ability to produce H_2_O_2_ when compared to other honey types with the same dilution [17]. White and Subers [49] showed that the inhibition of *S. aureus* growth occurs only when glucose is added to filtered honey (glucose removed). Moreover, when adding dry honey to the dialysed one as a glucose source, a much higher inhibition is exhibited when compared to artificial glucose, due to honey’s low level of free water. This was one of the first major experiments that exhibited honey’s osmotic effect due to its high total sugar concentration, as an advantageous property towards antimicrobial activity.

Extensive work in the last century confirms that honey releases enough H_2_O_2_ to provide significant antibacterial activity. The concentrations of H_2_O_2_ found in honey fluctuate greatly depending on the degree of its dilution, its botanical and geographical source, and its production and storage conditions. Commonly, H_2_O_2_ levels detected in various honey types range from 0.5 to 2.5 mM [17,50]. Roth et al. [51] comprehensively demonstrated H_2_O_2_ levels in 90 honey samples diluted to 14% *v*/*v* and incubated for 1 h, showing levels of H_2_O_2_ ranging between 0 and 2.12 mM. These data agree with Bang et al. [52], who showed concentrations between 1 and 2 mM in several varieties of New Zealand honey. The importance of incubation time was also shown in these assays, with rewarewa honey achieving 3.65 mM, its maximum concentration of H_2_O_2_ after 24 h, whereas ling heather honey reached its maximum level after only 4 h.

Dustmann [53] observed that the absolute levels of H_2_O_2_ present in any type of honey are determined by the corresponding levels of GOx and catalase. H_2_O_2_ production is directly proportional to GOx activity and inversely proportional to catalase levels. Catalase is a natural constituent of honey and catalyses the decomposition of H_2_O_2_ into water and oxygen. GOx levels are similar in most honeys across the world, as this enzyme is produced by the bees themselves. Nevertheless, as catalase is a plant-derived enzyme, its presence in the honey is dependent on the quantity and source of pollen collected by the bees and the subsequent catalase activity [54].

#### 2.1.2. Cytotoxicity Mechanism of Hydrogen Peroxide

Noticeable differences are recognised in the degree of the inhibitory effect of honey when tested against different bacterial strains. Dustmann [43] observed a more evident inhibitory activity against *Staphylococus aureus* and *Sarcina lute*, whilst *Streptococcus* spp., *Salmonella* spp., *Pseudomonas* and *Proteus* were less affected. These variations can be attributed not only to the different H_2_O_2_ levels present in different honeys, but also to the effectiveness of H_2_O_2_ mechanisms of cytotoxicity against each bacterial strain. H_2_O_2_ itself is not antimicrobial; the antibiotic effect occurs due to reactive hydroxyl free radicals originating from the catalytic action of traces of metal ions from the pathogen cells [55].

Imlay and Linn [56] identified two action modes of H_2_O_2_ against *Escherichia coli*, both being concentration-dependent. As observed, low H_2_O_2_ concentrations (1–2 mM) presented optimum conditions for killing bacteria through DNA damage. This mode represents a major portion of H_2_O_2_ toxicity and is facilitated by a Fenton reaction that employs H_2_O_2_, DNA-bound iron, and a steady source of reducing equivalents to generate active forms of hydroxyl radicals [57].

Highly reactive hydroxyl radicals are produced from the interaction of superoxide (O_2_^−^) radicals and H_2_O_2_, as proposed by Haber and Weiss [58]:

(1) $$O_{2}{}^{•-}+H_{2}O_{2}\to {\mathrm{OH}}^{•}+O_{2}+{\mathrm{OH}}^{-}$$

Even though several transition metals are able to catalyse this reaction, the major mechanism within cells (in vivo) is based on the iron-catalysed Haber–Weiss reaction, which uses Fenton chemistry [59,60]:

(2) $${\mathrm{Fe}}^{2+}+H_{2}O_{2}\to {\mathrm{Fe}}^{3+}+{\mathrm{OH}}^{•}+{\mathrm{OH}}^{-}$$

The antimicrobial effects of H_2_O_2_ can be attributed to these hydroxyl radicals and other oxygenated species acting as powerful oxidising agents, reacting with lipids, proteins, and nucleic acids [60]. Oxidative stress targeted towards nucleic acids leads to the production of modified bases such as 8-hydroxyguanine, urea, hydroxymethyl urea, and thymine glycol, whereas the modification of deoxyribose sugar, a component of DNA, can cause strand breaks [61]. This corresponds to mode-one of antibacterial action of H_2_O_2_, as proposed by Imlay and Linn [56].

The second mode is associated with higher H_2_O_2_ concentrations, where the antimicrobial efficacy depends less on concentration, but is directly proportional to exposure time, causing damage to several cellular targets [56]. This is evidenced by the production of protein carbonyls formed by the oxidation stress of arginine, proline, or lysine [61]. Furthermore, exposure of *E. coli* to H_2_O_2_ resulted in the oxidation of proteins including alcohol dehydrogenase E, elongation factor G, DnaK, OppA, enolase, OmpA, and F_0_F_1_-ATPase [62]. This protein damage, assessed by the quantification of carbonyl content, can be attributed to the loss of bacterial viability; as the carbonyl content increases, the viability decreases.

A minimum concentration of H_2_O_2_ is required to be effective in oxidative damage. A study of bacterial cultures supplemented with a H_2_O_2_ solution showed that the lowest H_2_O_2_ concentration capable of DNA degradation was 2.5 mM [50]. Interestingly, honey was shown to achieve DNA degradation with H_2_O_2_ levels detected below 2.5 mM. Hence, it can be concluded that H_2_O_2_ plays a crucial role in bacterial growth inhibition and DNA degradation through oxidative damage, but its activity is modulated by other non-peroxide factors [50].

### 2.2. Non-Peroxide Antimicrobial Activity

Several honey varieties have been demonstrated to have antibiotic efficacy even after catalase has decomposed H_2_O_2_ into water and oxygen [40]. Hence, it is widely accepted that there are non-peroxide factors that contribute to antimicrobial activity.

#### 2.2.1. Osmotic Effect

White et al. [13] produced a detailed study on roughly 504 samples of American honey and honeydew from 47 states. They concluded that honey’s moisture, or water presence, is low, averaging 17.2% by weight (ranging between 13.4 and 22.9%). Moreover, they showed that the main components of honey are fructose (38.19%), dextrose (31.28%), sucrose (1.31%), maltose (7.31%), and higher sugars (1.50%). This effectively allows honey to be classified as a super-saturated solution of sugars. Undiluted honey can inhibit bacteria growth as this high sugar concentration of honey exerts osmotic pressure on bacterial cells, which causes dehydration by transporting water out of bacterial cells through osmosis [63].

The strong interaction between these sugars with water molecules prevents the abundance of free water molecules available for microbes to grow [17]. The amount of free water molecules in honey is defined as the water activity (a_w_) [64]. Honey’s a_w_ ranges between 0.5 and 0.65 [65,66,67]. The lower the moisture content, the lower its average a_w_. Still, honeys with similar moisture content can show significant differences in their water activities. Honey crystallisation predominantly results in the crystallisation of glucose; as water molecules in honey are bound to sugars via hydrogen bonding this crystallisation frees the water molecules bound to glucose, thus increasing honey’s a_w_ [67]. Hence, a liquid honey sample has lower a_w_ than the same sample in a crystallised condition [64,67].

Amor [12] reported that for ripened honey, fermentation cannot occur if moisture is below 17.1%, as the a_w_ would be too low to promote the growth of any microbial species. The efficacy of inhibition in microorganism growth through this water withdrawal (osmotic) effect varies depending on the species in question. For instance, a_w_ required for microorganism development is around 0.70 for mould, 0.80 for yeast, and 0.90 for bacteria [67]. Generally, it is expected that honeys with low a_w_ are the most effective against pathogens with low tolerance to these conditions.

Nonetheless, there are microbial species with an extraordinary ability to withstand low a_w_ that are still vulnerable to honey’s inhibition potency. An example is *Staphylococcus aureus*, which, even though it can grow in a_w_ as low as 0.83, is still sensitive to the antimicrobial activity of honey [68]. Fungi are generally more tolerant to low a_w_ than bacteria but are still susceptible to honey’s antimicrobial activity [17].

#### 2.2.2. Acidity and pH

Honey is acidic with an average pH of 3.91, but can range between 3.4 and 6.1 [13] This acidity predominantly arises from gluconolactone/gluconic acid, originating from the enzymatic breakdown of glucose.

Prior to the full understanding of H_2_O_2_ release, the osmotic effect of sugars and honey’s low pH was believed to be the most significative characteristic that granted its antibiotic efficacy [69]. However, in a study with 81 honey samples, a linear correlation between bacterial inhibition and acidity was identified [70]. It was also shown that some honeys with pH above 5, such as honeydew- and chestnut-derived honeys, are effective in preventing bacterial growth [70]. In addition, several experiments with gluconolactone/gluconic acid solutions showed no bacterial inhibition when used in concentrations equivalent to that found in honey samples presenting a significant inhibitory effect [17]. Despite the majority of experimental studies being conducted with honey of neutral acidity, in clinical applications such as in wounds, bacteria are in contact with honey that is less diluted and more acidic, thus presenting high inhibition effects. This agrees with Bogdanov’s [70] conclusion that the main antimicrobial effect comes from honey’s acidity.

The effect of honey’s pH on the inhibition of microbial growth depends on the microbial strain. In general, moulds can grow in lower pH than yeasts, and yeasts can grow in lower pH than bacteria [71]. Honey is a successful antimicrobial agent against many animal pathogens with an optimum pH for growth ranging between 7.2 and 7.4, being particularly effective against common wound-infecting strains such as *Salmonella* species, *E. coli*, *Pseudomonas aeruginosa*, and *Streptococcus pyogenes*, which have a minimum pH for growth of 4.0, 4.3, 4.4, and 4.5, respectively [17]. Conversely, the low pH does not offer a substantial contributing factor to honey’s antimicrobial activity against fungi. For instance, the minimum pH for the growth of *Aspergillus niger* and *Candida albicans* is 1.2 and 2.2, respectively [71].

#### 2.2.3. Phenolic Content

Phenolic compounds originating from plant nectar have been proposed as important components for honey’s non-peroxide antimicrobial activity. When studying the inhibitory efficacy of plant extracts against bacteria, polyphenols are widely mentioned as one of the main contributing factors [72]. They are one of the most important groups of compounds in plants, with at least 8000 different known structures [73,74]. Phenols can be chemically defined as substances that have an aromatic ring bound with one or more hydroxyl groups. In food, their presence has a substantial effect on oxidative stability and microbiological safety [73].

The polyphenols identified in honey, used as potential chemical markers to determine its botanical origin and authenticity, are phenolic acids (benzoic and cinnamic acids) and flavonoids (flavonols, flavones, and flavanones) [75]. They are essential contributors to honey’s colour, taste, and health benefits.

Honey is produced by bees from the collection of natural products (e.g., flower volatiles, nectar, and pollen) and their own processed compounds (e.g., beeswax, propolis, and honey itself) [54]. Honey’s phenolic composition is fundamentally similar to propolis’, a resinous substance commonly known as “bee glue”, which is normally used by bees for the construction of the beehive. Capillary zone electrophoresis of propolis extracts has detected twelve different flavonoids, pinocembrin, acacetin, chrysin, rutin, luteolin, kaempferol, apigenin, myricetin, catechin, naringenin, galangin, and quercetin, as well as two phenolic acids, caffeic acid and cinnamic acid [14,76].

Metzner et al. [77] attributed the antibacterial activity of propolis to flavonoids and other components such as substituted benzoic and cinnamic acids. Honey presents a similar mechanism, as shown by Metzner et al. [78], who demonstrated that the flavonoids present in honey are derived from propolis rather than pollen as the main source. It has been suggested that the flavonoids’ antibiotic activity is due to the inhibition of bacterial energy metabolism, DNA gyrase, and cytoplasmic membrane function [79].

However, Scheller et al. [80] found that the individual components of propolis did not show antibiotic properties, and this activity was only observed when combined, suggesting that the flavonoids present in propolis do not significantly contribute to antimicrobial activity when acting individually. Since flavonoids are 1000 times less abundant in honey than in propolis, one can expect that flavonoids, benzoic acids and cinnamic acids may support honey’s antibacterial activity, but this contribution is small compared to that of H_2_O_2_ [54,78]. Moreover, the activity of honey may be the result of the combination of different phenolics, as opposed to individual phenols [81]. The phenolic content may simply be an enhancer of honey’s antimicrobial efficacy. For example, Al-Waili et al. [82] studied the addition of propolis to honey, observing a significant improvement in the antimicrobial effect against *S. aureus* and *E. coli* due to an increase in the phenolic content. This reduced the minimum inhibitory concentration (MIC) value of raw honey without propolis by up to half. Additionally, the antimicrobial activity of propolis has been shown to be greater against Gram-positive bacteria than Gram-negative [83]. This may also be applicable to the phenolic content of honey.

Honeydew produces a higher H_2_O_2_ content compared to blossom honey [84]. Furthermore, honeydew honey contains a higher content of phenolic acids and flavonoids, which have antioxidant and pro-oxidant properties [84]. When polyphenols are in the presence of transition metal ions (e.g., Fe and Cu) and peroxides, they can function as pro-oxidants by accelerating hydroxyl radical formation and oxidative strand breakage in DNA [84]. Whether polyphenols show antioxidative or antibacterial properties depends mainly on the pH value. In alkaline conditions (pH 7.0–8.0), polyphenols can display pro-oxidative properties, inhibiting microbial growth. Moreover, it is assumed that polyphenols at concentrations found in honeydew honey could support the production of considerable amounts of H_2_O_2_ via a non-enzymatic pathway, contributing considerably to honey’s antimicrobial effect [84].

Hence, polyphenols at concentrations found in some honey types, such as honeydew, contribute significantly to honey’s antimicrobial activity in two ways: by directly producing H_2_O_2_, and by reducing Fe (III) to Fe (II), triggering the Fenton reaction, which creates more potent ROS such as hydroxyl radicals [84]. Bucekova et al. [85] demonstrated that the overall antimicrobial activity of blossom honeys was strongly correlated with H_2_O_2_. However, there was no correlation between GOx content and H_2_O_2_ levels, suggesting that the phenolic content was contributing to the H_2_O_2_ production.

The most important flavonoids extracted from honey are acacetin, apigenin, chrysin, kaempherol, naringenin, pinobanksin, pinocembrin, and quercetin (Figure 2). Relevant reported antimicrobial phenolic acids extracted from honey include caffeic acid, ferulic acid, 4-hydroxybenzoic acid, and vanillic acid [71,73,75]. Other important phenolic compounds are present in several honeys, but their presence varies greatly depending on the floral and geographical source. 

#### 2.2.4. Defensin-1

Def-1, also historically referred to as royalisin, is an antibacterial peptide made of 51 amino acids that belongs to the defensin group of peptides [86]. Bee Def-1 mRNA has been detected in young worker bees’ hypopharyngeal gland. These bees then mature and age to be major honey producers, adding secretions from their hypopharyngeal glands to the collected nectar which includes Def-1 [87,88].

Kwakman et al. [87] showed that Def-1 in honey contributed to antibiotic action against *B. subtilis*. Bee Def-1 has potent antibacterial activity predominantly against Gram-positive bacteria such as *B. subtilis*, *S. aureus*, and *Paenibacillus*. Sojka et al. [89] demonstrated the crucial role of Def-1 in honey’s antibiofilm activity against wound-specific pathogens, especially *S. aureus*. The authors proposed two mechanisms of action against biofilm formation: by interfering with bacterial adhesion to a surface or in the early biofilm stage by inhibiting the growth of attached cells; and by altering the production of extracellular polymeric substances. Insect defensins in general have poor activity against Gram-negative bacteria [90]. However, recombinant Def-1 has been reported to have activity against Gram-negative bacteria, including *Pseudomonas aeruginosa* and *Salmonella enterica* [89]. 

Def-1 is present in all examined types of larval jelly and honey, including manuka honey, although amounts vary significantly [91]. The antibacterial importance of this peptide has spurred new methods for its quantification. For example, Valachova et al. [91] developed a polyclonal antibody-based competitive enzyme-linked immunosorbent assay to detect Def-1 and honeybee-derived proteins in honey, which could be a sensible approach for the verification of the authenticity of honey, and to rapidly screen the suitability of different honeys for medicinal purposes in terms of their potential for high antibacterial activity.

By neutralising Def-1, a significant reduction in antimicrobial activity of honey was displayed, confirming the important role of Def-1 as a non-peroxide antimicrobial agent [87]. Furthermore, neutralising H_2_O_2_, MGO, and Def-1 simultaneously, all antimicrobial activity ceased, suggesting that these are the most important factors responsible for the broad spectrum of honey’s bactericidal efficacy [87].

#### 2.2.5. Methylglyoxal

A study on the antibacterial properties of 345 samples of commercial unpasteurised honey from New Zealand showed that manuka honey, a monofloral honey derived predominately from the nectar of the *Leptospermum scoparium* (manuka) plant, holds superior antimicrobial efficacy over other honey sources [92]. H_2_O_2_ was removed from all samples through the addition of catalase, and manuka honey was one of the only two types showing activity in significant amounts, the other being a honey derived from *Echium vulgare* (vipers bugloss). This confirmed the presence of an important non-peroxide compound, subsequently identified as MGO [92,93,94]. 

MGO was identified by Weigel et al. [93] by studying the storage of commercial *Leptospermum* honeys and the development of 1,2-dicarbonyl compounds. The rate and efficiency of production of these compounds is related to the storage temperature [95]. These compounds, along with 5-hydroxymethylfurfural (HMF), are formed by reducing sugars in honey when they are heated through the Maillard reaction or caramelisation [96]. Suortti and Malkki [97] investigated the antibacterial properties of heated glucose and fructose and established a direct relationship between the rise in temperature of these monosaccharides and the increase in the inhibitory activity against *Escherichia coli*. The authors also discarded HMF as responsible for this inhibition.

Mavric et al. [94] investigated the possibility that 1,2-dicarbonyl compounds were associated with honey’s non-peroxide antimicrobial activity. This study observed that manuka honey was high in MGO content, being up to 100 times the identified amount in conventional honeys. MIC studies were performed using MGO, glyoxal, and 3-deoxyglucosulose for the inhibition of the bacterial growth of *E. coli* and *S. aureus*. These MICs were compared to diluted honeys in water, and the results show that samples diluted to 80% *v*/*v* exhibited no inhibition, whilst manuka honey displayed clear antibiotic properties with concentrations as low as 15% *v*/*v*. This concentration corresponds to MGO concentrations of about 1.1 mM, which was previously confirmed as the MIC of neat MGO [94]. The distinct antibacterial activity of New Zealand manuka honey due to MGO is represented commercially by the “Unique Manuka Factor” (UMF).

MGO is a highly reactive α-dicarbonyl compound generally formed endogenously during glycolytic pathways in cells, and exogenously by the fermentation of carbohydrate-containing foods and drinks, the heat treatment of sugar compounds, and the degradation of lipids [81,98]. MGO has been reported in various foods in concentrations of 3–47 mg/kg [81]. In contrast, significantly higher concentrations are commonly found in commercially available manuka honey, ranging from 30 to 950 mg/kg (0.58–18.5 mM), as displayed in Table S1. Some of these manufacturers offer manuka honey with MGO concentrations that exceed 1200 mg/kg, but these are rare in large quantities.

MGO in manuka honey is generated by the non-enzymatic conversion of dihydroxyacetone (DHA), a saccharide found in high concentrations in the nectar of *Leptospermum* flowers. This conversion process occurs at a slow rate in the nectar; thus, fresh manuka honey contains low levels of MGO, whilst the high concentrations of MGO develops during storage at 37 °C [81,95,99]. Unlike many other types of honey, *Leptospermum* honeys maintain antimicrobial activity even when exposed to high temperatures [100].

The reported strong correlation between MGO levels in manuka honey and its potential for bacterial inhibition suggests that MGO is mainly responsible for manuka’s non-peroxide activity. Nevertheless, Kwakman et al. [101] demonstrated that after the neutralisation of MGO, manuka honey was inactive against *S. aureus* and was substantially reduced against *B. subtilis*. However, manuka honey retained full bactericidal activity against *E. coli* and *P. aeruginosa* due to unknown factors [101]. It is worth highlighting that H_2_O_2_ was not detected in the manuka samples studied. It can be concluded that MGO is a major bactericidal factor, but may not be fully responsible for manuka’s non-peroxide antimicrobial activity; further investigation is required to understand other potential factors.

MGO’s antibiotic activity can be attributed to alterations in bacterial fimbriae and flagella, which obstructs bacteria’s adherence and motility [22]. High concentrations of MGO (around 2 mM) can lead to the partial or even complete loss of fimbriae and flagella, as well as damage to cell membranes and the shrinking of bacterial cells [102].

Although MGO has been demonstrated as a potent antimicrobial; there is concern related to the use of manuka-based honey and dressings in diabetic patients [99]. The high concentrations of both 1,2-dicarbonyl compounds and advanced glycation end products (AGEs) in diabetic patients is linked to poor wound healing and vascular complications [103,104,105]. MGO is a reactive metabolite and powerful glycating agent and is a precursor to the formation of AGEs, both of which can significantly impact the healthy function of cells and tissues, especially in diabetic patients. Therefore, further studies and clinical trials are required to determine the efficacy and safety of manuka-based products, especially products with high concentrations of MGO, in diabetic patients with chronic wounds.

### 2.3. Antibacterial Activity

The antibacterial properties of honey are widely acknowledged and have been extensively reported for a wide range of bacterial strains, including chronic wound isolates (Table S2). The rising prevalence of antibiotic-resistant bacterial strains is a serious cause for concern; thus, the broad-spectrum antibacterial properties of honey offer a potential alternative solution to antibiotics for specific topical applications [2,3,4].

Table S2 displays a wide range of antibiotic properties for honey at varying dilutions, from different honeybee species and with different botanical sources. As mentioned in previous sections, H_2_O_2_, bee Def-1, and MGO (*Leptospermum* honeys) are generally honey’s main mechanisms of action. Nevertheless, the key contributor to bacterial inhibition depends on each honey’s physico-chemical properties, influenced by its botanical source, honeybee species, the entomological proteins included, and the inhibition efficacy is also specific to the strain affected. A recent study on Chinese samples demonstrated how variations in bee species and botanical sources lead to significant differences in pH, conductivity, free acid, lactone acid, hydroxymethylfurfural content, moisture, ash, fructose, glucose, sucrose, and maltose contents, and colour [106]. 

Another important factor that determines the bacterial inhibition efficacy is honey’s moisture content and dilution. Table S2 reports inhibition by highly diluted honey samples with MICs as low as 3.1% *v*/*v* against *S. aureus* [107,108,109,110], *S. epidermidis* [109], *E. coli* [109], and *P. aeruginosa* [109]. Less effective honeys present MICs as high as 50% *v*/*v* [110]. The antibacterial activity generally decreases along with the increasing moisture content of honey. Moisture content may vary significantly between honeys, even when harvested at the same location, at the same time [106]. 

The broad spectrum of antibiotic activity exhibited by honey includes drug-resistant organisms, e.g., vancomycin-resistant *Enterococcus faecalis* [107], *Enterococcus raffinosus* [107], and methicillin-resistant *Staphylococcus aureus* [107,110,111,112,113]. This has led to investigations of honey–antibiotic synergy, with promising results. The addition of honeydew showed a synergistic antibacterial effect with ampicillin against *E. coli*, showing a larger diameter of inhibition zones, compared to honeydew honey alone, and no zone of inhibition for ampicillin alone. Similarly, the combination of honeydew honey with gentamicin was also synergistic [114]. Moreover, the pairing of manuka honey with tetracycline exhibited an increased antimicrobial affect against *P. aeruginosa* and *S. aureus* [115]. Sub-inhibitory concentrations of honey have also reduced or eliminated resistance to antibiotics. For example, Medihoney used alongside rifampicin exhibited a higher sensitivity of rifampicin against laboratory *S. aureus* strain NCTC 8325 and both MRSA (RPAH18, IMVS67 and MW2) and non-MRSA (04-227-3567) clinical isolates set up in cation-adjusted Mueller–Hinton II Broth [116]. Sub-inhibitory concentrations of honey, with the addition of oxacillin, also resulted in the restored susceptibility of MRSA to oxacillin [117]. The synergistic action also has been demonstrated in enhanced biofilm disruption. Examples of this are combinations of vancomycin with manuka honey against *S. aureus*, gentamicin with manuka honey against *P. aeruginosa* [118], and Portuguese honey combined with phage therapy in *E. coli* biofilm destruction [119].

### 2.4. Anti-Fungal Activity

The increasing rate of fungal infections in community and hospital environments, along with the limited availability of effective antifungal agents, has led many researchers around the world to exploring traditional medicine routes, and honey has been receiving increased attention in the last decade [120].

Azoles are the most used antifungal class, particularly to treat *Candida* infections [121]. Examples include fluconazole, which is often chosen due to its low cost, low toxicity, and availability for oral administration. However, there is extensive evidence of several *Candida* species, such as the emergent and concerning *Candida auris* species, which has intrinsic and developed resistance to azole antifungals [121,122]. There are three less used classes of antifungal drugs, including polyenes, pyrimidine analogues, and echinocandins. Even though the spectrum of available antifungals has become wider in recent decades, the choice of adequate antifungal agent is still restricted due to the emergence of more resistant fungal species, drug availability in immunocompromised patients, drug interaction, the toxicity of agents, and the lack of suitable routes of administration [123,124].

Honey activity against fungal strains is summarised in Table S3. There is clear evidence that some honey types, such as jujube (*Ziziphus jujuba*), not only show antifungal properties, but also demonstrate the ability to inhibit the formation of *C. albicans* biofilms and disrupt previously formed biofilms [125]. Honey’s inhibitory effect on fungus has been attributed to its osmotic effect [126]. However, Molan [17] argued this claim by highlighting honeys that even with low sugar concentration had inhibited fungi, proving that honey does present antifungal action unrelated to osmotic conditions alone. Using four representative honey types, Irish et al. [127] reported clinically significant antifungal activity against clinical isolates of Candida species: *C. albicans*, *C. glabrata* and *C. dubliniensis*. Moreover, Katiraee et al. [128] showed antifungal activity against all 11 fungal strain isolates when using six types of Iranian monofloral honey samples including *Thymus vulgaris*, *Alfalfa*, *Citrus*, *Zizyphus*, *Astragalus*, and *Chamaemelum nobile*, and one Iranian multiflora honey. Additionally, their work showed that honey’s antifungal activity is equally effective against fluconazole-susceptible, dose-dependent, and resistant *Candida* strains. 

Honey’s antifungal activity, apart from H_2_O_2_ production, is linked to other factors such as polyphenols and acidity, which have a clear relation to antifungal efficacy but vary greatly depending on the honey’s origin. Anand et al. [129] have demonstrated that several phenolic and volatile compounds are also responsible for antifungal activity. The authors identified the most significant compounds based on their relation to reported antifungal efficacy from different honey sources. In the case of Agastache honey, the antifungal activity is attributed to estragole [1-methoxy-4-prop-2-enylbenzene], phenol-2,4-bis (1,1-dimethylethyl) [(3,5-di*tert*-butylphenoxy)-trimethylsilane], 2,4-di*tert*-butylphenol, and several benzaldehydes; these compounds were reported to be effective against different fungal species, namely *Trichophyton*, *Aspergillus*, *C. albicans*, and dermatophytes, respectively. For honeys with a *Leptospernum* origin (manuka and tea-tree), the major antifungal compound identified was acetanisole [1-(2-methoxyphenyl)ethenone]. *Leptospermum polygalifolium* ‘Super manuka’ honey exhibited methyl 3,5-dimethoxybenzoate as the key marker for antifungal activity of this specific honey type. This compound has also been reported to be effective against *Candida albicans* [130]. Other important compounds in *Leptospermum* honeys include linalool, acetanisole, and nonanal, which have been reported to be effective against *P. vulgaris* [131]. The presence of aromatic acids such as benzyl cinnamate, methyl cinnamate, caffeic acid, and terpenoids have also been attributed to the antifungal properties of some honeys, especially honey with high propolis content [132].

### 2.5. Antiviral Activity

There are limited reports on the efficacy of honey against viruses, but the available evidence encourages further research, particularly against new viruses that are immune to common antiviral agents.

Honey showed good antiviral properties against the *Rubella* virus activity when tested in vitro using infected monkey kidney cell cultures [133]. This underlines the relevance of honey as an important bioactive biomaterial for clinical applications, apart from its use in traditional medicine, as can be observed with its wide incorporation into cough syrups [133]. The UK National Institute for Health and Care Excellence (NICE) lists honey as one of the main choices of self-care treatments for acute cough, as they have evidence of some benefit for the relief of cough symptoms [134].

Honey has been proven effective when applied topically on recurrent labial and genital herpes lesions in 16 adult patients [135]. Furthermore, when compared to acyclovir, the most common antiviral treatment, honey was substantially superior in terms of mean duration of attacks and pain, occurrence of crusting, and mean healing time. It is important to note that the use of honey also completely remitted two cases of labial herpes and one case of genital herpes, and no related adverse events were reported. Al-Waili [135] attributed honey’s efficacy to its flavonoids, H_2_O_2_, and ascorbic acid. A recent randomised controlled trial with a much larger group (952 adults) suggests that New Zealand kanuka honey cream (90% medical-grade kanuka honey, 10% glycerine) may work as well as acyclovir as a topical treatment of herpes simplex labialis (HSL) [136]. The authors reported no statistically significant differences between these treatments [136]. 

Manuka and clover honeys exhibited antiviral activity in vitro against varicella zoster virus in a study aiming to find potential remedy for shingles, suggesting honey as a viable option for viral skin rashes [79]. Manuka honey was also shown to be effective against influenza virus in vitro, using Madin-Darby canine kidney cells as a model [137].

A definite correlation between honey’s composition and its antiviral activity has not yet been fully defined. However, based on the current data available, honey flavonoids are proposed as crucial for their efficacy against viruses [138,139]. This claim is based on the repeatedly reported inhibitory effect of some flavonoids commonly present in honeys, against various viruses such as human immunodeficiency virus (HIV) [138,139]. Research around this global epidemic is generally focused on the HIV-1 strain and its enzymes. Flavonoids such as chrysin and apigenin have been shown to prevent HIV-1 activation [139]. From these, chrysin attracts more attention as it presents the highest therapeutic index against HIV-1 among 21 natural flavonoids [138].

Flavonoids extracted from propolis, also present in honey, have been demonstrated to be highly active in inhibiting the replication of different types of herpes viruses (HSV) [140,141]. Moreover, these flavonoids reduced the replication of rotavirus and human coronavirus (OC43) [140].

The potency of antiviral activity has been shown to improve with a combination of flavones and flavonols, as it occurs naturally in propolis and honey, when compared to their individual compounds [138]. Similarly, synergism has been reported between flavonoids and other antiviral agents. For instance, quercetin and apigenin potentiate the antiviral effects of acyclovir against HSV, suggesting that honey could be used to obtain enhanced activity of commercially available antiviral medications [138].

### 2.6. Commercial Medical-Grade Honey

Since honey has antimicrobial properties, most microbes cannot grow or survive in it. However, some bacterial strains such as *Bacillus* and *Clostridium* can form endospores, the dormant form of vegetative bacteria, which are highly resistant to low a_w_ and other physical and chemical influences [142,143]. Therefore, these bacterial strains, particularly *Bacillus*, may survive in raw honey after contamination, often via bees. These vegetative bacteria cannot multiply in honey, but can still be found in high numbers due to recent contamination [142]. Due to this, it is advised that young infants do not eat honey. *Clostridium botulinum*, which can cause gangrene or wound botulism, is occasionally detected, which agrees with reports of infant botulism due to honey consumption [142]. Like other bee-derived products, honey is also contaminated by pesticides, antibiotics, heavy materials, and radioactive isotopes [142]. Ingesting honey from unknown sources and with undefined safety may be a hazard to health. Hence, when clinical applications are intended, medical-grade honey (MGH) must be sterilised, typically via gamma irradiation, to eliminate any bacterial spores that are potentially present. This also highlights the importance of regulations from national and international food and health organisations regarding honey production, handling, and safety [81,142].

In an effort to promote clear MGH standards, Hermanns et al. [144] provided five minimum requirements for MGH: (1) organic, non-toxic, and free of contaminants; (2) free of pathogens through standardised gamma radiation; (3) safe to implement in medical therapies; (4) follows strict production and storage standards; and (5) complies with physicochemical criteria required for wound care products. 

Gamma radiation at a dose of 10 kGy has been proved to be an effective sterilisation method, eliminating bacterial contamination without any negative effects on the antibacterial and antibiofilm activity of honeydew honey [145]. Moreover, this dose does not affect the content of Def-1 in honeydew honey. Doses up to 30 kGy still do not result in significant alterations in the antibacterial and antibiofilm activity. Nevertheless, doses of gamma radiation above 10 kGy have been shown to significantly reduce Def-1 content [145].

Since regulated honey-based wound care products can be perceived as costly, table honey found in supermarkets is sometimes considered as a cheaper substitute. However, table honey has shown to be less effective at destroying pathogens in wounds and contains more microbial spores when compared to medical-grade honey [146]. This was demonstrated by Cooper and Jenkins (2009) by comparing the antibiotic potency of 18 table honeys to a sample of *Leptospermum* MGH. Higher antimicrobial activity was observed in the MGH, as well as the presence of a wide range of microbial species in the table honeys, whereas MGH was sterile [100]. MGH has been proved to be effective and safe to use on wound environments, even for patients with diabetes, as there is no evidence of a significant effect on blood sugar levels [146]. The current recommended application period for MGH treatments is two weeks [146]. 

Predominately, MGHs have been focused around *Leptospermum*-derived honeys such as manuka or jelly bush, as these non-peroxide honeys maintain antimicrobial activity when exposed to high temperatures and catalase [100]. Their dilute concentrations demonstrated consistent efficacy towards antibiotic-sensitive bacteria and antibiotic-resistant bacteria, both being equally susceptible [100]. Most MGHs come from New Zealand, taking advantage of their unique manuka flower. Manuka honey is distinguished from other types by its two unique fluorescence signatures. Bong et al. [147] showed that one of the fluorescence markers is due to leptosperin, a *Leptospermum* nectar-derived compound now widely used for the recognition of manuka honey authenticity.

Additional research over the last few years showed that over 200 signature compounds, in combination, are unique to authentic manuka honey [148]. A shortlist of these compounds is used to determine its genuineness. A key compound identified was leptosperin, which is chemically stable even when stored for prolonged periods over 37 °C [147]. Moreover, its relevance to manuka honey identification comes from its complexity. Since it is hard to synthetically manufacture, it is assumed to be only present in genuine manuka honey [148]. Furthermore, DHA and MGO can also be used to distinguish manuka honey. Studies on the presence of these compounds are currently used to support the UMF quality trademark, with a higher UMF number reflecting higher MGO content, and hence greater antimicrobial activity.

Currently, the UMF Honey Association (Auckland, New Zealand) oversees all use of their quality trademark by ensuring compliance with license agreements, industry standards, and regular sample checks from the marketplace [149]. There are currently more than 100 beekeepers, producers, and exporters accredited to display the UMF quality trademark on manuka honey products, which covers over 80% of all New Zealand manuka honey exports. These commercially available products display a number on their label, as established by the UMFHA grading system. This number directly represents the presence of the combination of key signature markers: leptosperin, MGO, DHA, and HMF [149].

An alternative MGO-only grading system verifies and certifies the natural MGO content present in the honey due to its natural variance. This system simply states how much MGO is present: for example, an MGO of 400+ means that the honey contains at least 400 mg/kg of MGO [150]. However, it needs to be noted that MGO can also be produced synthetically. Therefore, companies such as Comvita (Paengaroa, New Zealand) have opted to utilise a dual grading system with both MGO and UMF, for authenticity and further antibacterial assurance for customers [151].

Even though these grading systems reflect the expected non-peroxide activity, studies have shown that they may not completely reflect the product’s antimicrobial efficacy at the time of use. For instance, Girma et al. [152] found significantly lower antimicrobial activity at UMF15+ honey when compared to 5+ and 10+ honeys, with lower potency against Gram-negative bacteria when compared to staphylococcal pathogens. This shows that additional specialised tests are required for complex applications. Furthermore, it also shows the complexity of honey and how much more research is required to fully understand its properties. 

There are several routes of administration and products inspired/based on MGH. In England and Wales alone, there are about ten suppliers of medical-grade honey for the NHS Supply Chain, distributed in different applications and presentations [153]. The Clinical and Product Assurance team of the NHS Supply Chain ensures that these products are safe and provide demonstrable benefits to patients. Table 1 provides a list of some commercially available MGH-based or honey-inspired products for use in wound care, as well as their indications and clinical evidence.

## 3. Honey as a Wound Healing and Tissue Regenerative Agent

Honey’s ability to prevent wound infections and promote wound healing through its natural antimicrobial properties (H_2_O_2_ production, osmotic effect, polyphenols, etc.) and by acting as a physical barrier to the wound site has been extensively explored [16,17,27,28,33,61,70,71,182]. Honey’s antimicrobial properties are crucial for the body’s response to tissue damage. Protein-digesting enzymes produced by bacteria are harmful to tissues and are detrimental to the growth factors and extracellular matrix (ECM) produced by the body as it attempts to stimulate tissue regeneration [16,183,184]. Moreover, the reduction in oxygen availability, due to bacteria consumption, compromises tissue growth [185]. Thus, the elimination of bacteria within the wound site can promote tissue regeneration.

**Figure 4:**
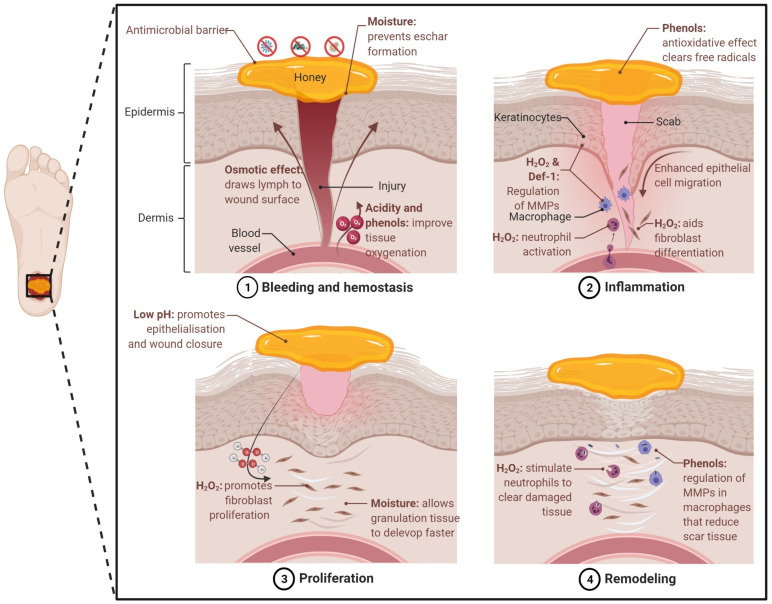
Key factors of honey that contribute to wound healing across all four healing phases. Created with BioRender.com (accessed on 23 February 2022).

In addition, honey also has properties that promote the regeneration of damaged tissue and wound healing. These properties are multi-factorial and associated with key aspects of the material such as moisture, pH, sugar content, ROS generation, and the anti-inflammatory effect. All of these aspects contribute to the four stages of the wound healing process: haemostasis (blood clotting), inflammation, proliferation/epithelialisation, and tissue remodelling (Figure 4).

*Moisture*: Although honey has a low water activity (‘free’ water), it provides a moist environment to the wound bed. This moist environment effectively provides a barrier that prevents eschar formation (dead tissue) and mitigates dermal necrosis, often observed in wounds exposed to air. The importance of moisture for wound healing has been widely demonstrated. Winter et al. [186] reported that epithelisation occurs faster, and a scab is avoided on skin wounds that are kept moist under a dressing, in contrast to wounds exposed to air. Svensjo et al. [187] further supported this claim and showed that granulation tissue develops faster in moist conditions, when compared to dry, and even wet conditions. Moreover, the moist wound surface enhances the migration of epidermal cells, as opposed to migration under the scab. An additional benefit of applying honey is the osmotic effect and subsequent drawing of water and lymph to the wound environment, which aids the oxygenation and nutrition of damaged tissue [27]. Furthermore, the creation of a mixture of diluted honey and drawn lymph under the dressing prevents it from adhering to the wound bed, minimising the risk of tearing newly formed tissue when changing the dressing [16,27].

*pH and sugar content:* The high sugar content contributes to the high osmolarity of honey and has been suggested to provide localised nutrition to the wound site [188]. The application of honey provides a low pH environment, which has been shown to promote epithelialisation and wound closure [189]. This low pH also may reduce the activity of proteases and limit ECM removal [190]. Moreover, this acidification promotes oxygen dissociation from haemoglobin, the Bohr effect, which results in improved tissue oxygenation [189]. However, studies have also shown that acidic conditions can prevent wound closure and re-epithelialisation [191,192]. However, the sustained and relatively low pH levels in these studies may not be applicable when using honey-based products.

*Reactive oxygen species*: Historically, the production of ROS in cells was seen as a consequence of an anaerobic environment. Moreover, ROS such as H_2_O_2_ have been classed as harmful and responsible for molecular damage such as DNA mutation and protein oxidation. Hence, it was believed that it was imperative for cells to eliminate these oxidising species [193].

However, a more important and complex role for ROS in biological functions such as wound healing and growth regulation has been demonstrated [20,194]. The production of H_2_O_2_ is induced when cells are exposed to epidermal growth factor. The ROS produced activates signalling pathways that lead to cell proliferation and differentiation. Furthermore, a clear correlation between the increase in ROS production and increase in mitogenic rate has been identified [194,195]. Furthermore, Love et al. [34] demonstrated that there is a continuous release of H_2_O_2_ during tail regeneration in *Xenopus* tadpoles with amputated tails. This showed that injury-induced ROS production is a crucial regulator of tissue regeneration.

Subsequently, the role of H_2_O_2_ generation in honey is a crucial aspect of its potential use in tissue regeneration applications. ROS levels influence the different stages of wound healing [20]. For example, H_2_O_2_ released from honey has been shown to stimulate the proliferation of fibroblasts when used in a time- and dose-dependent manner [196]. However, the authors also show that prolonged exposure to high concentrations of H_2_O_2_ can exhibit a cytotoxic effect. Additionally, honey’s phenolic content and its antioxidant properties can counteract this toxic effect, rendering protection to cells and enhancing their growth [196,197]. Furthermore, honey has the potential to supply the levels of H_2_O_2_ required for the Wnt signalling pathway, which is widely implicated in regenerative processes [34,193,194]. ROS can aid in tissue regeneration through the activation of neutrophil protease [198,199]. This enzyme lays inactive inside neutrophil granules until stimulated by the inactivation of its inhibitor. This required inhibitor inactivation occurs as a result of ROS oxidation, hence releasing neutrophil protease to carry out the proteolytic removal of damaged wound tissue, which can potentially simplify debridement in chronic wounds. The regulation of matrix metalloproteinases (MMPs), crucial to the healing process in chronic wounds, can be influenced by honey [200,201,202,203,204]. ROS in skin wounds have been shown to promote the activation of nuclear factor erythroid derived 2-like 3 (Nrf2), which, in turn, increased the activity of MMPs in fibroblasts [205]. Both the up- and downregulation of MMPs in keratinocytes have been observed when cultured with honey and honey-derived flavonoids, which provides contradictory conclusions [202,203]. The use of different honey types may contribute to the discrepancies, and the amount of ROS generated has not been adequately quantified. ROS may be involved in the regulation of MMPs; however, further research is required.

The H_2_O_2_ released from honey to the wound site will influence multiple wound healing pathways and have complex effects on aspects of cellular behaviour, including proliferation, signalling, metabolism, and migration. Maintaining a low level of ROS is likely key to promoting tissue regeneration and wound healing, as the high and excessive production of ROS can lead to oxidative stress and impaired wound healing [206].

*Defensin-1*: The antibacterial peptide, Def-1, has been shown to be responsible for promoting re-epithelialisation in vivo in a study using royal jelly [86]. The presence of Def-1 elevates the keratinocyte production of MMP-9 and enhances keratinocyte migration, resulting in a significant increase in wound closure rates.

*Anti-inflammation*: Honey’s anti-inflammatory ability also plays a crucial role in tissue regeneration. During haemostasis, blood flow can be restricted through the capillaries (ischaemia) causing oxygen starvation (hypoxia), along with a lack of nutrients, both of which are vital for cell proliferation, which is required to repair tissue damage [16]. In addition, the previously mentioned antioxidative effect attributed to honey’s high phenolic content also supports anti-inflammation effects. These compounds exhibit radical scavenging properties due to the high reactivity of their hydroxyl radicals, clearing the free radicals formed due to inflammation [84,207,208]. This antioxidative effect has further been found to counter necrosis and reduce ischaemia on burns [209,210]. On the other hand, in weakly alkaline conditions (pH 7.0–8.0), honey’s phenolic acids and flavonoids demonstrate oxidative potential. Pro-oxidative phenols accelerate hydroxyl radical formation and H_2_O_2_ production, enhancing honey’s antimicrobial and anti-inflammatory effects [84,208]. 

### Honey for Tissue Engineering Applications

**Figure 5:**
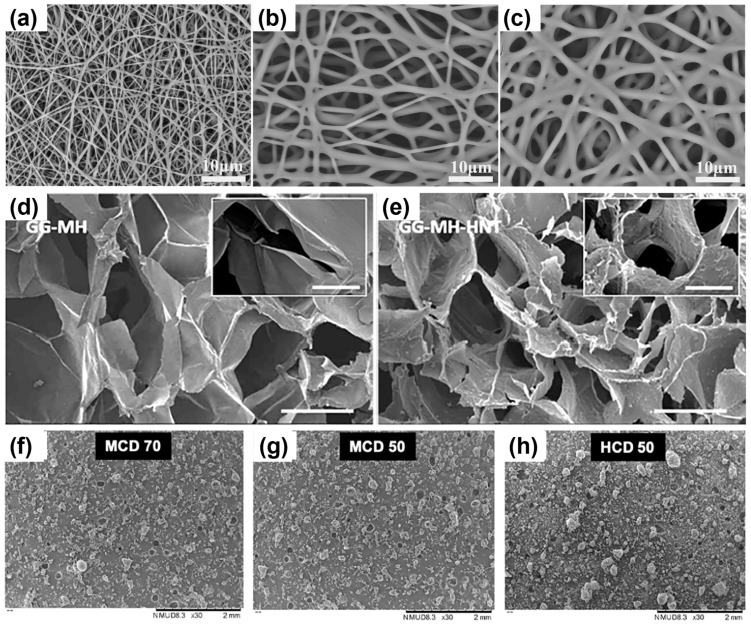
Honey-containing scaffolds. Scanning electron microscopy images of electrospun fibres containing (**a**) 0%, (**b**) 30%, and (**c**) 70% manuka honey [212]; (**d**) gellan gum hydrogels with 2% manuka honey and (**e**) reinforced with clay halloysite nanotubes [240]; and freeze-dried powders using methylated-β-cyclodextrin and (**f**) 70% or (**g**) 50% SurgihoneyRO™ and (**h**) (2-hydroxypropyl)-β-cyclodextrin with 50% SurgihoneyRO™ [254]. Reproduced with permission from Elsevier.

Honey and tissue-engineered honey-based products have been explored to treat acute and chronic wounds by direct application, as a dressing, or in combination with other materials. When used as a topical agent it requires a secondary wound dressing such as gauze to protect the wound and contain the honey at a specific location, as the honey can leak away from the wound. The difficulty in the delivery and sustained release of the active ingredients of honey has facilitated the development of new strategies. Tissue-engineered scaffolds containing honey offer a potential route to precisely deliver and sustain honey at the site of wound healing and in other tissue regeneration applications [29,32,33]. Electrospinning [211,212,213,214,215,216,217,218,219,220,221,222,223,224,225,226,227,228,229,230,231,232,233,234,235], hydrogels and cryogels [219,220,236,237,238,239,240,241,242,243,244,245,246,247,248,249,250], foams [251,252], films [253], powders [254], cements [255], and bioinks [256,257] have been utilised to fabricate honey-based scaffolds (Figure 5). 

Electrospinning is the most commonly used approach to fabricate honey-based scaffolds due to its versatility in material and solvent compatibility, high surface area and porosity, allowing the loading of bioactive agents (e.g., nanoparticles, drugs, and growth factors), and its ability to produce nanofibres that can mimic the ECM. The non-woven fibrous meshes, produced through electrostatic acceleration and the elongation of a polymer jet and subsequent solvent evaporation or melt solidification, are widely explored as wound dressings [258]. Honey has been used in combination with polymers such as polyvinyl alcohol (PVA), cellulose acetate (CA), and polycaprolactone (PCL) to fabricate electrospun meshes. Schuhladen et al. [218] produced electrospun nanofibrous PCL and methylcellulose (MC) meshes containing manuka honey and bioactive glass. The presence of MGO in the manuka acted as a novel crosslinker for the MC. The meshes showed improved wettability, bioactivity, and cell viability and migration. However, the meshes showed no noticeable antibacterial properties against *S. aureus* or *E. coli*, which was attributed to the low manuka concentration used. The therapeutic properties of honey can be complemented by using additional natural bioactive agents. Gaydhane et al. [227] developed electrospun multi-layered PVA/CA fibres loaded with honey and curcumin, which had anti-inflammatory properties. The composite meshes showed enhanced antioxidant properties and moderate antibacterial activity. Alternatively, Ghalei et al. [226] developed a polylactic acid mesh containing honey and an nitric oxide donor, S-nitroso-N-acetyl-penicillamine, a potent antibacterial. The meshes showed sustained nitric oxide release for up to 48 h, a synergistic antibacterial effect with a 95% reduction in *S. aureus* and *E. coli*, and high cell viability and proliferation. The ability of honey to promote wound healing is a key factor in the use of honey in dressings. Yang et al. [212] fabricated a silk fibroin electrospun mesh containing manuka. The meshes showed significant bacterial inhibition, especially at a high manuka loading concentration, whilst supporting cell proliferation. An in vivo wound study in a mouse model showed a similar healing and closure rate by day 12 compared to a commercially available wound dressing, AquacelAg (ConvaTec Inc., Reading, UK).

Hydrogels, crosslinked polymer networks swollen by water, are widely explored in tissue engineering and drug delivery applications due to their aqueous and porous three-dimensional structure, mimicking the native ECM, which allows the encapsulation of biomolecules and enables cell attachment, proliferation, and migration [31,259,260]. The ability to precisely tune the physiochemical, mechanical, and biological properties of the hydrogel enables a wide range of applications to be considered. For example, Bonifacio et al. [239] developed a gellan gum and manuka hydrogel with tuneable mechanical properties and release profiles of MGO depending on the type of cation crosslinker and presence of an inorganic material. Biofilms composed of clinical isolates of *S. aureus* and *S. epidermidis* cultured with the hydrogel showed a significant reduction in viability. The hydrogels were cytocompatible and exhibited chondrogenic differentiation. Subsequently, further investigation using silica, bentonite, and halloysite fillers showed improved mechanical properties [240]. The hydrogels were able to inhibit bacterial growth in an infected scaffold implanted into an in vivo mouse model; additionally, the silica improved this inhibition. PVA-based hydrogels which are biocompatible, water-soluble, highly swelling, and non-toxic have been explored, with honey showing antibacterial properties. A manuka and PVA hydrogel crosslinked using sodium tetraborate and containing 80% honey in the dry state was developed by Tavakoli and Tang [236]. The hydrogel exhibited the sustained release of honey for over 24 h, low adhesion in a model after 24 h swelling, and the significant inhibition of *S. aureus* but negligible inhibition of *E. coli*. An alternative crosslinking method for PVA is freeze–thawing, or cryogelation, explored by Santos et al. [249] in the development of a multi-layer hydrogel with graded honey concentrations. The samples showed negligible inhibition against *S. aureus*, attributed to the low manuka concentration used. Shamloo et al. [243] fabricated PVA hydrogels by freeze–thawing, which contained gelatin, chitosan, and honey. PVA by itself has poor bioactivity; thus, adding chitosan and gelatin provides a haemostatic agent and cell-binding motifs, respectively. The antibacterial inhibition against *P. aeruginosa* and *S. aureus* increased with a higher concentration of honey and showed higher inhibition than a hydrogel dressing for burns (Burn Tec, KikGel Ltd., Ujazd, Poland). The hydrogels were cytocompatible and in an in vivo rat model increased the rate of wound closure and formed well-defined epidermal and dermal tissue with increased expression of collagen.

Hixon et al. [219] compared the properties of silk fibroin electrospun meshes, hydrogels, and cryogels containing manuka. The use of a single material, silk, was to elucidate how the structural properties of the scaffold influenced bacterial inhibition. The electrospun scaffolds had a higher inhibition of *S. aureus* than the hydrogel or cryogels. This was attributed to the high surface area of the fibres allowing the rapid release of the manuka and the flat mesh structure having a greater contact area with the bacteria. This demonstrates the importance of scaffold design for the intended application.

An alternative approach by Hall et al. [254] is the development of an absorbent and in situ gelling powder containing SurgihoneyRO™ (Matoke Holdings Ltd., Abingdon, UK), a commercially available engineered honey with demonstrated antimicrobial and wound healing properties [18,19,178,179,180]. A starch-based drying agent combined with freeze-drying and milling was used to produce a powder (particle size ~200 µm). Sodium polyacrylate was incorporated to allow in situ gelation, which was observed after <1 min in response to a volume of simulated wound exudate forming a hydrogel barrier that filled the defect. The powders showed production of H_2_O_2_ (~30 µmol g^−1^ at the peak) for up to 8 days. This resulted in the inhibition of the growth of *P. aeruginosa*, *E. coli*, and *S. aureus*. Additionally, high cell viability and comparable cell proliferation to a cell-only control was observed when cultured with different powder concentrations. Subsequently, Hall et al. [255] explored the development of a calcium sulphate cement containing SurgihoneyRO™ (Matoke Holdings Ltd., Abingdon, UK) for orthopaedic applications. The production of H_2_O_2_ in the cements peaked at 24 h and the inhibition of *S. aureus* and *P. aeruginosa* growth was comparable to a dose of gentamicin.

The versatility and variety of approaches using honey in scaffolds shows the drive to reformulate honey into innovative delivery systems for both antimicrobial and tissue-regenerative applications. For example, a novel approach is the use of bioprinting to develop alginate scaffolds [256] and pectin patches [257] containing honey. The predominant application areas are wound dressings, but new areas such as cartilage [239,240] and bone [255] are being explored, which demonstrates the potential of honey-based scaffolds outside the traditional clinical uses.

However, the majority of studies lack characterisation for the presence of GOx in the processed honey-based scaffold or the generation of H_2_O_2_. This is key for the peroxide-based antimicrobial properties and the modulation of cell behaviour. Furthermore, the harsh processing steps utilised in the development of the scaffolds (e.g., high temperatures, use of solvents, crosslinking steps, and sterilisation protocols) may denature the GOx, rendering it inactive. Additionally, prolonged contact with water during processing can prematurely activate the GOx and initiate the production of H_2_O_2_. However, other honey antimicrobial and bioactive compounds may remain active, especially MGO in manuka-honey-based scaffolds.

## 4. Conclusions and Research Challenges

Honey is a complex material with a huge range of varieties depending on botanical and geographical origin, species of bee, and production methods. This variety leads to the differing levels and consistency of antimicrobial efficacy. Although the main antimicrobial effect common to all honeys is the release of H_2_O_2_, the presence of other active compounds such as MGO and Def-1 has been confirmed to have antimicrobial properties and can vary between varieties. A key challenge is ascertaining and determining the antimicrobial efficacy of the large number of polyphenols and other compounds present in honey and furthermore their specific impact on biological pathways in tissue regeneration. These compounds offer a bank of potential antimicrobial and regenerative agents that can be utilised to combat antibiotic resistance and aid in tissue healing. By isolating the key efficacious compounds, synthetic biomimetic honey-inspired biomaterials can be developed that are not reliant on specific honey producers and the risks involved in the processing (e.g., sterilisation, storage, and transport), determining authenticity (e.g., adulteration with other materials), safety (e.g., presence of fungal/bacterial spores and pesticides), and material consistency of natural honey. These honey-inspired biomaterials can be formulated to contain only the active compounds required, such as GOx, MGO, and Def-1, and designed to mimic honey by maintaining a moist environment with a low pH and high sugar levels in order to be effective as a wound dressing, for example.

Apart from the H_2_O_2_, MGO, and Def-1 antimicrobial properties, honey has also been shown to restore the susceptibility of antibiotic-resistant bacteria to antibiotics [116,117,261,262]. Methicillin-resistant *Staphylococcus aureus* (MRSA) exposed to subinhibitory concentrations of manuka honey in combination with oxacillin acted synergistically to resensitise MRSA to oxacillin and inhibit growth [117]. The combination of honey and conventional antibiotics acting synergistically to inhibit the growth of bacteria and biofilm formation is promising. Using honey as an adjunct to the primary therapy can expand the therapeutic window, lower the drug dosage, and reduce antibiotic resistance through complementary killing mechanisms. This is especially relevant in chronic wound environments with bacterial biofilms as they are far more tolerant to antibiotics.

The production of H_2_O_2,_ the main antimicrobial factor, via GOx is an oxygen-negative process. Furthermore, the subsequent biological decomposition of H_2_O_2_ into water and oxygen in vivo by the enzyme catalase only recovers stoichiometrically half the oxygen, thus depleting the microenvironment of oxygen, which limits the activity of GOx. This two-enzyme system, GOx and catalase, has been used to create hypoxic environments for cellular studies due to the net consumption of oxygen [263]. However, this potentially presents a problem for honey-based and -inspired products, as the oxygen consumption may lead to hypoxia in the wound environment, with negative consequences in healing. Additionally, a major issue in the tissue engineering of tissue-scale constructs is vascularisation and the supply of oxygen and nutrients during either in vitro culture or after implantation in vivo [264]. However, bioreactor perfusion systems and the inclusion of vascular endothelial growth factor (VEGF) can aid in oxygenation and promotion of blood vessel formation. There is a requirement for oxygenation at the beginning of new tissue formation and during wound healing; thus, alternative approaches incorporating oxygen-releasing materials such as calcium peroxide, magnesium peroxide, and H_2_O_2_ (direct use) have been considered. For example, Erdem et al. [265] bioprinted a gelatin methacryloyl bioink including calcium peroxide that maintained an oxygenated environment for up to 7 days and enhanced fibroblast viability under hypoxic conditions. Harnessing the production and delivery of oxygen in biomaterials can aid in the wound healing process by preventing necrosis and promoting fibroblast proliferation [266,267,268]. Furthermore, these materials used in conjunction with honey can prevent the development of a hypoxic environment and provide an oxygen source to maintain GOx enzyme activity, thus providing a supply of H_2_O_2_.

A major complication of utilising honey in wound treatments is the mode of delivery (application and ease of use) and efficacy, maintaining the direct contact of the honey with the wound bed. Increased volumes of honey are required to preload traditional dressing materials, which may also lead to dressing failure, leakage, and the loss of antimicrobial activity (efficacy), and potentially result in further peri-wound skin complications. Furthermore, this can reduce the ability of dressing materials to effectively absorb wound exudate, leading to dressing failure and increases in the frequency of dressing changes, clinical visits, and dressing costs. Subsequently, innovations affording the honey and its active ingredients sustained and direct wound bed contact may prove both clinically and cost effective. This may necessitate reformulating honey into hydrogels, electrospun fibres, and granules, in addition to further developments of impregnated wound dressings. These approaches can allow a more targeted delivery mechanism of active ingredients and sustained antimicrobial activity whilst addressing clinical cost efficacy, ease of use, and complication prevention. The additional clinical benefits of honey, including maintaining a lower pH, high sugar, and moisture balance, should also be considered.

The role of H_2_O_2_ in both antibacterial activity and tissue regeneration raises the question of the required concentration and time required to achieve both. High levels of H_2_O_2_ can be an effective antimicrobial, but cytotoxic to cells and tissues. On the other hand, lower levels that may promote positive cellular regenerative responses can be detrimental to antimicrobial efficacy, especially in chronic wound environments where biofilms are present. A balance between the level of H_2_O_2_ needed for antimicrobial activity and tissue regeneration is needed. This may be achieved by the temporal control of H_2_O_2_ production and release to have a sustained antimicrobial effect [269]. The concentration could shift with time from an initial high release rate for antimicrobial efficacy to a lower, more sustained concentration to promote tissue regeneration. Controlling this precisely, especially in a wound environment, is a significant challenge.

Honey is a key biomaterial and source of inspiration for advanced wound care therapies. However, further in vitro and in vivo studies are required to elucidate specific mechanisms of both antimicrobial action and wound healing. These studies should ascertain the effectiveness of honey against bacterial biofilms in addition to the typical planktonic and colony studies. Furthermore, the definition of standards should be a key priority; for example, the use of appropriate controls when comparing the effectiveness of honey or honey-based dressings and scaffolds. The most relevant and clinical gold standard for wound management should be used, rather than a gauze dressing.

Honey as a natural biomaterial has exceptional medical properties, which have been utilised by humans throughout history. This review provides a comprehensive overview of the antimicrobial properties of honey and its application in the wound environment. The role of honey in wound healing and tissue regeneration is discussed. The past few decades have seen a renewed interest in this material due to new approaches in wound care management, an increase in antimicrobial resistance, and a renewed appreciation of honey’s antimicrobial and wound healing properties. Subsequently, honey has been approved for wound care applications (Table 1) and shown to offer improved symptomatic relief of upper respiratory tract infections [270]. This offers an alternative to conventional antimicrobial drugs, which are increasingly becoming ineffective due to antimicrobial resistance, and provides a new tool for clinicians and nurses.

The acidity, osmolarity, H_2_O_2_ generation, and range of additional compounds (e.g., MGO, Def-1, and phenolics) present in honey provide a broad-spectrum antimicrobial effect, which enables honey to essentially have an indefinite shelf-life. This repository of compounds may have immense medical benefit, so further research is required to identify and ascertain their specific antimicrobial contributions. The multifactorial and synergistic antimicrobial effect of honey, which makes antimicrobial resistance or immunity difficult to impossible, provides lessons in the design of new antimicrobials and treatment regimens that can combat the rise of antimicrobial-resistant pathogens. The additional benefit of honey to wound healing, through increasing epithelisation, promoting cell migration, stimulating cells, and facilitating debridement, further highlights the importance of honey as a key treatment in wound care.

A deeper understanding of the molecular composition of honey and the subsequent biological interactions is required to fully appreciate the potential biomedical applications available. Additionally, honey is not the only bee product that has potential biomedical applications, with honeybee venom showing anti-cancer properties, demonstrating that the humble bee has a lot more to offer to biomedical science [271].

## Figures and Tables

**Table 1:** Commercially available honey-based wound healing products.

Product	Manufacturer	Description	Indications	Mechanism of Action	Ref.	Clinical Evidence
Activon^®^ Manuka Honey Tube	Advancis Medical	100% medical-grade manuka honey	Any wound type but especially sloughy, necrotic, and malodorous wounds, including: pressure ulcers, leg ulcers, diabetic ulcers, surgical wounds, burns, graft sites, infected wounds, cavity wounds and sinuses	Debrides necrotic tissue; can be used in dressings or directly into cavities.	[154]	Inhibition of in vitro formation of clinically important Gram-positive bacteria biofilms [155]. Blistering and cellulitis on a type 2 diabetic patient; paediatric burn; foot ulceration; grade 5 sacral wound [154]
Activon^®^ Tulle	Advancis Medical	Knitted viscose mesh dressing impregnated with 100% manuka honey	Granulating or shallow wounds, good when debriding or de-sloughing small areas of necrotic or sloughy tissue	Creates a moist healing environment, eliminates wound odour, and provides antibacterial action	[154]	Overgranulated grade 3 and 4 pressure ulcers; extensive leg cellulitis; venous ulcer; chronic wound infections; necrotic foot [154]
Algivon^®^ Plus	Advancis Medical	Reinforced alginate dressing impregnated with 100% manuka honey	Pressure, leg and diabetic ulcers, surgical wounds, burns, graft sites and infected wounds. Ideal for wetter wounds	Absorbs exudate. Debrides, removes slough, and reduces bacterial load	[154]	Chronic wounds [156]; burn wound management [157]
Algivon^®^ Plus Ribbon	Advancis Medical	Reinforced alginate ribbon impregnated with 100% manuka honey	Cavities, sinuses, pressure ulcers, leg ulcers, diabetic ulcers, surgical wounds, burns, graft sites, and infected wounds	Absorb exudates. Debrides, removes slough, and reduces bacterial load	[154]	Autoamputation of fingertip necrosis [158]
Aurum^®^ ostomy bags	Welland Medical Ltd.	Medical-grade manuka honey added to the hydrocolloid	Stoma care	Kills bacteria, suppresses inflammation, and stimulates the growth of cells to promote healthy skin around the stoma	[159]	Pyoderma gangrenosum around ileostomy [160]
L-Mesitran^®^ Border	Aspen Medical Europe Ltd.	Combined hydrogel and honey (30%) pad on a strong fixation layer	Chronic wounds, such as: pressure ulcers; superficial and partial-thickness burns; venous, arterial, and diabetic ulcers.	Exudate absorption. Donates moisture to rehydrate dry tissue. Antibacterial properties. Helps to maintain a moist wound environment	[161]	Paediatric minor burns and scalds [162]
L-Mesitran^®^ Hydro	Aspen Medical Europe Ltd.	Sterile, semi-permeable hydrogel dressing containing 30% honey with vitamin C and E, as well as an acrylic polymer gel and water, with a polyurethane film backing	Low to moderate exuding wounds, including: chronic wounds (pressure ulcers, venous and diabetic ulcers), superficial and acute wounds (cuts, abrasions and donor sites), superficial and partial-thickness burns (first- and second-degree), fungating wounds, acute wounds, e.g., donor sites, surgical wounds, cuts and abrasions	Donates moisture to rehydrate dry tissue. Antibacterial properties. Helps to maintain a moist wound environment	[161]	Paediatric minor burns and scalds [162]. Fungating wounds [163]
L-Mesitran^®^ Ointment	Aspen Medical Europe Ltd.	Ointment with 48% medical-grade honey, medical-grade hypoallergenic lanolin, oils, and vitamins	Superficial, acute, and chronic wounds. Superficial and partial-thickness burns. Fungating wounds (to help deodorise and debride). Colonised acute wounds and (postoperative) surgical wounds	Aids debridement and reduce bacterial colonisation	[161]	Skin tears; irritation and inflammation [163]
ManukaDress IG	Medicareplus International	Wound dressing made with 100% *Leptospermum scoparium* sterile honey from New Zealand. Non-adherent impregnated gauze	Leg and pressure ulcers, first- and second-degree burns, diabetic foot ulcers, surgical and trauma wounds	Osmotic activity that promotes autolytic debridement and helps maintain a moist wound environment	[164]	Burn management [165]. Difficult-to-debride wounds [166]. Necrotic pressure ulcer; recurrent venous leg ulceration [167]
Medihoney^®^ Antibacterial Honey	Derma Sciences—Comvita	100% sterilised medical-grade manuka honey	All types of wounds with low to moderate exudate, including: deep, sinus, necrotic, infected, surgic and malodorous wounds^®^	Creates an antibacterial environment (MGO). Autolytic debridement on sloughy and necrotic tissue. Removes malodour. Provides a moist environment.	[168]	Wound healing [169]; prevention of catheter-associated infections in haemodialysis patients [170]
Medihoney^®^ Apinate Dressing	Derma Sciences—Comvita	Calcium alginate dressing impregnated with 100% medical-grade manuka honey	Moderately to heavily exuding wounds such as: diabetic foot ulcers, leg ulcers, pressure ulcers (partial- and full-thickness), first- and second-degree partial-thickness burns, donor sites and traumatic or surgical wounds.	Promotes a moisture-balanced environment. Osmotic potential draws fluid through the wound to the surface. Low pH of 3.5–4.5.	[171]	Venous leg ulcers [172]
Medihoney^®^ Barrier Cream	Derma Sciences—Comvita	Barrier cream containing 30% medical-grade manuka honey	Use to protect skin from breakdown (e.g., skin damaged by irradiation treatment or in wet areas due to incontinence). Additionally, to prevent damage caused by shear and friction	Maintains skin moisture and pH.	[173]	Treatment for intertrigo in large skin folds [174]
Medihoney^®^ Antibacterial Wound Gel™	Derma Sciences—Comvita	Antibacterial wound gel: 80% medical-grade manuka honey with natural waxes and oils	Surface wounds with low to moderate exudate and partial- and full-thickness wounds, including burns, cuts, grazes, and eczema wounds	Creates a moist, low-pH environment. Cleans the wound through osmotic effect. Reduces the risk of infection (MGO)	[175]	Reduction in incidence of wound infection after microvascular free tissue reconstruction [176]
SurgihoneyRO™	Matoke Holdings Ltd.	Antimicrobial wound gel utilising bioengineered honey to deliver Reactive Oxygen^®^ (RO™)	Infected, chronic (diabetic foot, pressure, and leg ulcers) and acute (surgical, traumatic and abrasions wounds, cuts, burns, donor and recipient sites) wounds	Controlled release of hydrogen peroxide release for antimicrobial activity. Promotes debridement and new tissue growth	[177]	Prevention of caesarean wound infection; prevention/eradication of bacterial colonies in dressing oncology long vascular lines; ulcers, surgical wounds and trauma wounds [178,179,180]. In vitro activity against biofilm-producing clinical bacterial isolates [181]

## Supplementary Materials

The following supporting information can be downloaded at: https://www.mdpi.com/article/10.3390/pharmaceutics14081663/s1, Table S1: Commercially available manuka honey and MGO levels available. Table S2: Gram-positive and Gram-negative bacterial strains inhibited by different honey types, their corresponding minimum inhibitory concentration (MIC) values (lowest concentration of a chemical compound that prevents the visible growth of a bacterium), and their suggested mechanism of action. Table S3: Fungal strains inhibited by different honey types, their corresponding MIC values, and their mechanism of action, as suggested by the authors. References [19,107,108,109,110,111,112,113,125,127,128,129,272,273,274,275,276,277,278,279,280,281,282,283,284,285] are cited in Supplementary Materials.
