# Supplementary material for: Honey: An Advanced Antimicrobial and Wound Healing Biomaterial for Tissue Engineering Applications
Source: Pharmaceutics. 2022 Aug 10;14(8):1663. doi: 10.3390/pharmaceutics14081663 (PMC9414000; doi:10.3390/pharmaceutics14081663)
Supplement: Supplementary file 1 [file pharmaceutics-14-01663-s001.zip › pharmaceutics-1843345-supplementary.pdf]

Review

Honey: An Advanced Antimicrobial and Wound Healing Biomaterial for Tissue Engineering Applications

Joel Yupanqui Mieles, Cian Vyas, Enes Aslan, Gavin Humphreys, Carl Diver and Paulo Bartolo

Supplementary Tables

Table S1. Commercially available manuka honey and MGO levels available.

Manufacturer/Brand	MGO [mg/kg]	Reference
Comvita*	83+ to 829+ (UMF® 5+ to 20+)	[1]
Egmont Honey*	83+ to 829+ (UMF® 5+ to 20+) Limited editions of UMF27+	[2]
Manuka Doctor	30+ to 940+	[3]
Manuka Health	115+ to 950+	[4]
Manuka Pharm	40+ to 340+	[5]
New Zealand Manuka Group/Melora*	83+ to 829+ (UMF® 5+ to 20+)	[6]
Optima/ Pure Gold	85+ to 525+	[7]

* Manufacturers with UMF/dual grading system. UMF= Unique Manuka Factor – Grading system directly proportional to the amount of MGO, Leptosperin, dihydroxyacetone (DHA), and 5-hydroxymethylfurfural (HMF).

Table S2. Gram-positive and Gram-negative bacterial strains inhibited by different honey types, their corresponding minimum inhibitory concentration (MIC) values (lowest concentration of a chemical compound that prevents visible growth of a bacterium) and suggested mechanism of action.

GRAM-POSITIVE BACTERIA						
**Strain inhibited**	**Honey Type**	**Honey/plant source (species)**	**Origin**	**MIC (% v/v)**	**Mechanism of action**	**Ref.**
Coagulase-negative Staphylococci	Manuka (MGO 263+)	*Leptospermum scoparium*	New Zealand	9.1*	Methylglyoxal	[8]
	Tualang	*Koompassia excelsa*, Multifloral by *Apis dorsata* bees	Malaysia	7.2*	Hydrogen peroxide	[8]
Enterococcus faecalis vancomycin-sensitive	Artificial	-	-	29.7	Osmolarity and acidity	[9]
	Chestnut	*Aesculus*	Italy	25	Osmolarity, acidity and hydrogen peroxide	[10]
	Honeydew	*(Abies Alba)*	Poland	15	High total amount of polyphenolic compounds (flavonoids)	[11]
	Lime tree	*Tilia*	Italy	50	Osmolarity, acidity and hydrogen peroxide	[10]
	Manuka (MGO 250+)	*Leptospermum scoparium*	New Zealand	15	Methylglyoxal, high amount of polyphenolic compounds (flavonoids)	[11]
	Manuka (MGO 696+)	*Leptospermum scoparium*	New Zealand	4.9	Methylglyoxal	[9]
	Multiflora	Pasture	New Zealand	9.7	Hydrogen peroxide	[9]
	Rhododendron	*Rhododendron*	Italy	25	Osmolarity, acidity and hydrogen peroxide	[10]
Enterococcus faecalis vancomycin-resistant	Artificial	-	-	29.5	Osmolarity and acidity	[9]
	Manuka (MGO 696+)	*Leptospermum scoparium*	New Zealand	3.8	Methylglyoxal	[9]
	Multiflora	Pasture	New Zealand	5.6	Hydrogen peroxide	[9]

Enterococcus raffinosus vancomycin-resistant	Artificial honey	-	-	29	Osmolarity and acidity	[9]
	Manuka (MGO 696+)	*Leptospermum scoparium*	New Zealand	4.9	Methylglyoxal	[9]
	Multiflora	Pasture	New Zealand	9	Hydrogen peroxide	[9]
Staphylococcus aureus (MSSA)	Agastache	*Agastache Rugosa*	Australia	6.3	Methyl syringate, phenyllactic acid and hydrogen peroxide	[12]
	Buckwheat	*Fagopyrum esculentum* Moench	Poland	3.1	Hydrogen peroxide, polyphenols and antioxidants	[13]
	Chestnut	*Aesculus*	Italy	6.3	Hydrogen peroxide, polyphenols and antioxidants	[13]
	Chestnut and pine	*Castanea* and *Pinus*	Greece	3.1	Hydrogen peroxide and antibacterial proteinaceous compounds	[14]
	Dandelium	*Taraxacum*	Italy	25	Osmolarity, acidity and hydrogen peroxide	[10]
	Honeydew	*(Abies Alba)*	Poland	3.1	Hydrogen peroxide, polyphenols and antioxidants	[13]
	Jarrah	*Eucalyptus marginata*	Australia	12.5	Hydrogen peroxide	[12]
	Jelly bush	*Leptospermum polygalifolium*	Australia	12.5	Methylglyoxal	[12]
	Lime tree	*Tilia*	Poland	3.1	Hydrogen peroxide, polyphenols and antioxidants	[13]
	Lime tree	*Tilia*	Italy	25	Osmolarity, acidity and hydrogen peroxide	[10]
	Madrones	*Arbutus*	Greece	6.3	Hydrogen peroxide	[14]
	Manuka (medical grade)	*Leptospermum scoparium*	New Zealand	3.1	Methylglyoxal	[10]
	Manuka (MGO 250+)	*Leptospermum scoparium*	New Zealand	20	Methylglyoxal, high amount of polyphenolic compounds (flavonoids)	[11]
	Manuka (MGO 263+)	*Leptospermum scoparium*	New Zealand	9.1*	Methylglyoxal	[8]

Manuka (MGO 400+)	*Leptospermum scoparium*	New Zealand	15	Methylglyoxal, high amount of polyphenolic compounds (flavonoids)	[11]
Manuka (MGO 550+)	*Leptospermum scoparium*	New Zealand	6.3	Methylglyoxal	[14]
Manuka (MGO 971+)	*Leptospermum scoparium*	New Zealand	6.3	Methylglyoxal, phenyllactic acid and methyl syringate	[12]
Multiflora	-	Poland	1.6	Polyphenols and antioxidants	[13]
Multiflora	-	Kazakhstan	3.1	Hydrogen Peroxide, polyphenols and antioxidants	[13]
Multiflora	-	Italy	12.5	Osmolarity, acidity and hydrogen peroxide	[10]
Multiflora	Herbs	Greece	3.1	Hydrogen peroxide	[14]
Multiflora (*A. mellifera)*	Multifloral native forest, by Africanized *A. mellifera* bees	Brazil	10.6*	Phenolic content	[15]
Multiflora (*T. angustula*)	Typical native fores with *B. dracunculifolia,* *by T. angustula* bees	Brazil	12.8*	Phenolic content	[15]
Pine	*Pinus*	Greece	3.1	Hydrogen peroxide	[14]
Rhododendron	*Rhododendron*	Italy	25	Osmolarity, acidity and hydrogen peroxide	[10]
Sunflower	*Helianthus*	Greece	3.1	Hydrogen Peroxide and antibacterial proteinaceous compounds	[14]
Super manuka (MGO 400+)	*Leptospermum polygalifolium*	Australia	12.5	Phenyllactic acid, hydrogen peroxide and methyl syringate	[12]
Tea tree	*Leptospermum lanigerum & Leptospermum scoparium*	Australia	6.3	Hydrogen peroxide, phenyllactic acid and Methyl syringate	[12]
Thyme	*Thymus*	Cyprus	6.3	Hydrogen peroxide	[14]
Tualang	*Koompassia excelsa,* Multifloral by *Apis dorsata* bees	Malaysia	12.7*	Hydrogen peroxide	[8]

Staphylococcus aureus Methicillin-resistant (MRSA)	Agastache	*Agastache Rugosa*	Australia	9.1*	Methyl syringate, phenyllactic acid and hydrogen peroxide	[12]
	Artificial honey	-	-	>30	Osmolarity and acidity	[9]
	Chestnut	*Aesculus*	Italy	25	Osmolarity, acidity and hydrogen peroxide	[10]
	Dandelium	*Taraxacum*	Italy	25	Osmolarity, acidity and hydrogen peroxide	[10]
	Honeydews	-	Italy	25	Osmolarity, acidity and hydrogen peroxide	[10]
	Jarrah	*Eucalyptus marginata*	Australia	18.1*	Hydrogen peroxide	[12]
	Jelly bush	*Leptospermum polygalifolium*	Australia	2.3*	Methylglyoxal	[12]
	Lime tree	*Tilia*	Italy	25	Osmolarity, acidity and hydrogen peroxide	[10]
	Manuka (medical grade)	*Leptospermum scoparium*	New Zealand	12.5	Methylglyoxal	[10]
	Manuka (MGO 1200+)	*Leptospermum scoparium*	New Zealand	12.5	Methylglyoxal	[16]
	Manuka (MGO 263+)	*Leptospermum scoparium*	New Zealand	9.1*	Methylglyoxal	[8]
	Manuka (MGO 696+)	*Leptospermum scoparium*	New Zealand	3	Methylglyoxal	[9]
	Manuka (MGO 971+)	*Leptospermum scoparium*	New Zealand	4.5*	Methylglyoxal, phenyllactic acid and methyl syringate	[12]
	Multiflora	Pasture	New Zealand	3.1	Hydrogen peroxide	[9]
	Multiflora	-	Italy	12.5	Osmolarity, acidity and hydrogen peroxide	[10]
	Rhododendron	*Rhododendron*	Italy	25	Osmolarity, acidity and hydrogen peroxide	[10]
	Super manuka (MGO 400+)	*Leptospermum polygalifolium*	Australia	9.1*	Phenyllactic acid, hydrogen peroxide and methyl syringate	[12]
	Tea tree	*Leptospermum lanigerum & Leptospermum scoparium*	Australia	2.3*	Hydrogen peroxide, phenyllactic acid and Methyl syringate	[12]

	Tualang	*Koompassia excelsa*, Multifloral by *Apis dorsata* bees	Malaysia	8.2*	Hydrogen peroxide	[8]
	Ulmo 90	*Eucryphia cordifolia*	Chile	6.3	Hydrogen peroxide	[16]
Staphylococcus epidermidis	Buckwheat	*Fagopyrum esculentum* Moench	Poland	3.1	Hydrogen Peroxide, polyphenols and antioxidants	[13]
	Chestnut	*Aesculus*	Italy	6.3	Hydrogen Peroxide, polyphenols and antioxidants	[13]
	Honeydew	-	Poland	6.3	Hydrogen Peroxide, polyphenols and antioxidants	[13]
	Lime tree	*Tilia*	Poland	3.1	Hydrogen Peroxide, polyphenols and antioxidants	[13]
	Multiflora	-	Poland	1.6	Polyphenols and antioxidants	[13]
	Multiflora	-	Kazakhstan	3.1	Hydrogen Peroxide, polyphenols and antioxidants	[13]
Streptococcus agalactiae	Manuka (MGO 263+)	*Leptospermum scoparium*	New Zealand	12.7*	Methylglyoxal	[8]
	Tualang	*Koompassia excelsa*, Multifloral by *Apis dorsata* bees	Malaysia	10.9*	Hydrogen peroxide	[8]
Streptococcus pyogenes	Manuka (MGO 263+)	*Leptospermum scoparium*	New Zealand	7.2*	Methylglyoxal	[8]
	Tualang	*Koompassia excelsa*, Multifloral by *Apis dorsata* bees	Malaysia	7.2*	Hydrogen peroxide	[8]

GRAM-NEGATIVE BACTERIA

Strain inhibited	Honey Type	Honey/plant source (species)	Origin	MIC (% v/v)	Mechanism of action	Ref.

Acinetobacter baumannii	Manuka (MGO 263+)	*Leptospermum scoparium*	New Zealand	9.1*	Methylglyoxal	[8]
	Tualang	*Koompassia excelsa,* Multifloral by *Apis dorsata* bees	Malaysia	7.2*	Hydrogen peroxide	[8]
Enterobacter cloacae	Manuka (MGO 263+)	*Leptospermum scoparium*	New Zealand	16.3*	Methylglyoxal	[8]
	Tualang	*Koompassia excelsa,* Multifloral by *Apis dorsata* bees	Malaysia	18.1*	Hydrogen peroxide	[8]
Escherichia coli	Agastache	*Agastache Rugosa*	Australia	6.3	Methyl syringate, phenyllactic acid and hydrogen peroxide	[12]
	Buckwheat	*Fagopyrum esculentum* Moench	Poland	6.3	Hydrogen Peroxide, polyphenols and antioxidants	[13]
	Chestnut	*Aesculus*	Italy	12.5	Hydrogen Peroxide, polyphenols and antioxidants	[13]
	Chestnut	*Aesculus*	Italy	25	Osmolarity, acidity and hydrogen peroxide	[10]
	Dandelium	*Taraxacum*	Italy	25	Osmolarity, acidity and hydrogen peroxide	[10]
	Honeydew	-	Poland	12.5	Hydrogen Peroxide, polyphenols and antioxidants	[13]
	Honeydews	-	Italy	12.5	Osmolarity, acidity and hydrogen peroxide	[10]
	Jarrah	*Eucalyptus marginata*	Australia	25	Hydrogen peroxide	[12]
	Jelly bush	*Leptospermum polygalifolium*	Australia	6.3	Methylglyoxal	[12]
	Lime tree	*Tilia*	Poland	6.3	Hydrogen Peroxide, polyphenols and antioxidants	[13]
	Lime tree	*Tilia*	Italy	25	Osmolarity, acidity and hydrogen peroxide	[10]
	Manuka	*Leptospermum scoparium*	New Zealand	12.5	Methylglyoxal	[10]
	Manuka (MGO 1200+)	*Leptospermum scoparium*	New Zealand	12.5	Methylglyoxal	[16]
	Manuka (MGO 250+)	*Leptospermum scoparium*	New Zealand	25	Methylglyoxal, high amount of polyphenolic compounds (flavonoids)	[11]

	Manuka (MGO 263+)	*Leptospermum scoparium*	New Zealand	20	Methylglyoxal	[8]
	Manuka (MGO 400+)	*Leptospermum scoparium*	New Zealand	20	Methylglyoxal, high amount of polyphenolic compounds (flavonoids)	[11]
	Manuka (MGO 971+)	*Leptospermum scoparium*	New Zealand	6.3	Methylglyoxal, phenyllactic acid and methyl syringate	[12]
	Multiflora	-	Poland	3.1	Polyphenols and antioxidants	[13]
	Multiflora	-	Italy	25	Osmolarity, acidity and hydrogen peroxide	[10]
	Multiflora	-	Kazakhstan	12.5	Hydrogen Peroxide, polyphenols and antioxidants	[13]
	Rhododendron	*Rhododendron*	Italy	25	Osmolarity, acidity and hydrogen peroxide	[10]
	Super manuka (MGO 400+)	*Leptospermum polygalifolium*	Australia	25	Phenyllactic acid, hydrogen peroxide and methyl syringate	[12]
	Tea tree	*Leptospermum lanigerum & Leptospermum scoparium*	Tasmania, Australia	12.5	Hydrogen peroxide, phenyllactic acid and Methyl syringate	[12]
	Tualang	*Koompassia excelsa*, Multifloral by *Apis dorsata* bees	Malaysia	22.5	Hydrogen peroxide	[8]
	Ulmo 90	*Eucryphia cordifolia*	Chile	12.5	Hydrogen peroxide	[16]
Proteus mirabilis	Chestnut	*Aesculus*	Italy	25	Osmolarity, acidity and hydrogen peroxide	[10]
	Dandelium	*Taraxacum*	Italy	50	Osmolarity, acidity and hydrogen peroxide	[10]
	Honeydews	-	Italy	25	Osmolarity, acidity and hydrogen peroxide	[10]
	Lime tree	*Tilia*	Italy	25	Osmolarity, acidity and hydrogen peroxide	[10]
	Manuka	*Leptospermum scoparium*	New Zealand	25	Methylglyoxal	[10]
	Manuka (MGO 263+)	*Leptospermum scoparium*	New Zealand	16.3*	Methylglyoxal	[8]
	Multiflora	-	Italy	25	Osmolarity, acidity and hydrogen peroxide	[10]
	Rhododendron	*Rhododendron*	Italy	50	Osmolarity, acidity and hydrogen peroxide	[10]

	Tualang	*Koompassia excelsa*, Multifloral by *Apis dorsata* bees	Malaysia	16.3*	Hydrogen peroxide	[8]
Pseudomonas aeruginosa	Agastache	*Agastache Rugosa*	Australia	4.5*	Methyl syringate, phenyllactic acid and hydrogen peroxide	[12]
	Buckwheat	*Fagopyrum esculentum* Moench	Poland	3.1	Hydrogen Peroxide, polyphenols and antioxidants	[13]
	Chestnut	*Aesculus*	Italy	12.5	Hydrogen Peroxide, polyphenols and antioxidants	[13]
	Chestnut	*Aesculus*	Italy	25	Osmolarity, acidity and hydrogen peroxide	[10]
	Dandelium	*Taraxacum*	Italy	50	Osmolarity, acidity and hydrogen peroxide	[10]
	Eucalyptus	*Eucalyptus*	Algeria	12	-	[17]
	Honeydew	*(Abies Alba)*	Poland	>25	High total amount of polyphenolic compounds (flavonoids)	[11]
	Honeydew	-	Poland	12.5	Hydrogen Peroxide, polyphenols and antioxidants	[13]
	Honeydews	-	Italy	25	Osmolarity, acidity and hydrogen peroxide	[10]
	Jarrah	*Eucalyptus marginata*	Australia	9.1*	Hydrogen peroxide	[12]
	Jelly bush	*Leptospermum polygalifolium*	Australia	9.1*	Methylglyoxal	[12]
	Lime tree	*Tilia*	Poland	6.25	Hydrogen Peroxide, polyphenols and antioxidants	[13]
	Lime tree	*Tilia*	Italy	25	Osmolarity, acidity and hydrogen peroxide	[10]
	Manuka	*Leptospermum scoparium*	New Zealand	25	Methylglyoxal	[10]
	Manuka (MGO 1200+)	*Leptospermum scoparium*	New Zealand	12.5	Methylglyoxal	[16]
	Manuka (MGO 250+)	*Leptospermum scoparium*	New Zealand	>25	Methylglyoxal, high amount of polyphenolic compounds (flavonoids)	[11]
	Manuka (MGO 263+)	*Leptospermum scoparium*	New Zealand	12.7*	Methylglyoxal	[8]
	Manuka (MGO 400+)	*Leptospermum scoparium*	New Zealand	>25	Methylglyoxal, high amount of polyphenolic compounds (flavonoids)	[11]

	Manuka (MGO 550+)	*Leptospermum scoparium*	New Zealand	12.5	Methylglyoxal	[14]
	Manuka (MGO 971+)	*Leptospermum scoparium*	New Zealand	18.1*	Methylglyoxal, phenyllactic acid and methyl syringate	[12]
	Multiflora	Herbs	Greece	6.3	Hydrogen peroxide	[14]
	Multiflora	-	Poland	3.1	Polyphenols and antioxidants	[13]
	Multiflora	-	Kazakhstan	6.3	Hydrogen Peroxide, polyphenols and antioxidants	[13]
	Multiflora	-	Italy	25	Osmolarity, acidity and hydrogen peroxide	[10]
	Orange blossom	*Citrus sinensis*	Algeria	18	-	[17]
	Origan and clover	*Trifolium*	Greece	6.3	Hydrogen Peroxide and antibacterial proteinaceous compounds	[14]
	Rhododendron	*Rhododendron*	Italy	50	Osmolarity, acidity and hydrogen peroxide	[10]
	Super manuka (MGO 400+)	*Leptospermum polygalifolium*	Australia	9.1*	Phenyllactic acid, hydrogen peroxide and methyl syringate	[12]
	Tea tree	*Leptospermum lanigerum & Leptospermum scoparium*	Tasmania, Australia	4.5*	Hydrogen peroxide, phenyllactic acid and Methyl syringate	[12]
	Tualang	*Koompassia excelsa,* Multifloral by *Apis dorsata* bees	Malaysia	12.7*	Hydrogen peroxide	[8]
	Ulmo 90	*Eucryphia cordifolia*	Chile	12.5	Hydrogen peroxide	[16]
Salmonella Enteritidis	Honeydew	*(Abies Alba)*	Poland	>25	High total amount of polyphenolic compounds (flavonoids)	[11]
	Manuka (MGO 250+)	*Leptospermum scoparium*	New Zealand	>25	Methylglyoxal, high amount of polyphenolic compounds (flavonoids)	[11]
	Manuka (MGO 400+)	*Leptospermum scoparium*	New Zealand	25	Methylglyoxal, high amount of polyphenolic compounds (flavonoids)	[11]
Salmonella Typhimurium	Honeydew	*(Abies Alba)*	Poland	>25	High total amount of polyphenolic compounds (flavonoids)	[11]

	Manuka (MGO 250+)	*Leptospermum scoparium*	New Zealand	25	Methylglyoxal, high amount of polyphenolic compounds (flavonoids)	[11]
	Manuka (MGO 263+)	*Leptospermum scoparium*	New Zealand	12.7*	Methylglyoxal	[8]
	Manuka (MGO 400+)	*Leptospermum scoparium*	New Zealand	25	Methylglyoxal, high amount of polyphenolic compounds (flavonoids)	[11]
	Tualang	*Koompassia excelsa,* Multifloral by *Apis dorsata* bees	Malaysia	10.9*	Hydrogen peroxide	[8]
Shigella flexner	Manuka (MGO 263+)	*Leptospermum scoparium*	New Zealand	18.1*	Methylglyoxal	[8]
	Tualang	*Koompassia excelsa,* Multifloral by *Apis dorsata* bees	Malaysia	18.1*	Hydrogen peroxide	[8]
Stenotrophomonas maltophilia	Manuka (MGO 263+)	*Leptospermum scoparium*	New Zealand	7.2*	Methylglyoxal	[8]
	Tualang	*Koompassia excelsa,* Multifloral by *Apis dorsata* bees	Malaysia	7.2*	Hydrogen peroxide	[8]

* MIC values converted from %w/v to %v/v using the honey density of 1.38g/mL (lowest reported density), to report the highest possible MIC.

Table S3. Fungal strains inhibited by different honey types, their corresponding MIC values and mechanism of action suggested by the authors.

FUNGAL STRAINS

Strain inhibited	Honey Type	Honey source (species)	Origin	MIC (% v/v)	Mechanism of action	Ref.
Aspergillus flavus	Alfalfa	*Alfalfa*	Iran	25	High phenolic content: tannins, pectin substances, saponines, amines, triterpene glycosides, purines base, phytoestrogens, flavones, isoflavonoids	[18]
	Astragalus	*Astragalus*	Iran	35	Acidic pH, hydrogen peroxide and high osmolarity	[18]
	Chamaemelum	*Chamaemelum nobile*	Iran	55	Acidic pH, hydrogen peroxide and high osmolarity	[18]
	Citrus	*Citrus*	Iran	50	Acidic pH, hydrogen peroxide and high osmolarity	[18]
	Multiflora	Multifloral	Iran	35	Acidic pH, hydrogen peroxide and high osmolarity	[18]
	Thymus	*Thymus vulgaris*	Iran	35	High phenolic content: caravacrol, thymol and gamma terpinene	[18]
	Zizyphus	*Zizyphus*	Iran	55	Acidic pH, hydrogen peroxide and high osmolarity	[18]
Aspergillus fumigatus	Alfalfa	*Alfalfa*	Iran	25	High phenolic content: tannins, pectin substances, saponines, amines, triterpene glycosides, purines base, phytoestrogens, flavones, isoflavonoids	[18]
	Astragalus	*Astragalus*	Iran	40	Acidic pH, hydrogen peroxide and high osmolarity	[18]
	Chamaemelum	*Chamaemelum nobile*	Iran	55	Acidic pH, hydrogen peroxide and high osmolarity	[18]
	Citrus	*Citrus*	Iran	55	Acidic pH, hydrogen peroxide and high osmolarity	[18]
	Multiflora	Multifloral	Iran	35	Acidic pH, hydrogen peroxide and high osmolarity	[18]
	Thymus	*Thymus vulgaris*	Iran	35	High phenolic content: caravacrol, thymol and gamma terpinene	[18]
	Zizyphus	*Zizyphus*	Iran	60	Acidic pH, hydrogen peroxide and high osmolarity	[18]
Aspergillus niger	Acacia	*Acacia mangium* trees by *Apis mellifera*	Malaysia	15	-	[19]

	Alfalfa	*Alfalfa*	Iran	30	High phenolic content: tannins, pectin substances, saponines, amines, triterpene glycosides, purines base, phytoestrogens, flavones, isoflavonoids	[18]
	Astragalus	*Astragalus*	Iran	35	Acidic pH, hydrogen peroxide and high osmolarity	[18]
	Chamaemelum	*Chamaemelum nobile*	Iran	55	Acidic pH, hydrogen peroxide and high osmolarity	[18]
	Citrus	*Citrus*	Iran	55	Acidic pH, hydrogen peroxide and high osmolarity	[18]
	Kelulut	Malaysian stingless bee	Malaysia	10	-	[19]
	Multiflora	Multifloral	Iran	40	Acidic pH, hydrogen peroxide and high osmolarity	[18]
	Thymus	*Thymus vulgaris*	Iran	35	High phenolic content: caravacrol, thymol and gamma terpinene	[18]
	Tualang	*Koompassia excelsa,* Multifloral by *Apis dorsata* bees	Malaysia	25	-	[19]
	Zizyphus	*Zizyphus*	Iran	55	Acidic pH, hydrogen peroxide and high osmolarity	[18]
Candida albicans	A. mellifera	Multifloral, *Apis mellifera* bee	Mexico	40	Flavonoids cause shrinkage of cell membrane, loss of polarization, and damages on the cell walls of C. albicans.	[20]
	Acacia	*Acacia mangium* trees by A*pis mellifera*	Malaysia	15	-	[19]
	Agastache	*Agastache Rugosa*	Australia	29*	Phenolic compounds: Phenol, 2,4-bis(1,1-dimethylethyl), estragole and nonanoic acid and ethyl ester	[21]
	Alfalfa	*Alfalfa*	Iran	24.5	High phenolic content: tannins, pectin substances, saponines, amines, triterpene glycosides, purines base, phytoestrogens, flavones, isoflavonoids	[18]

Artificial honey	7.5 g sucrose, 37.5 g maltose, 167.5 g glucose, and 202.5 g fructose in 85 mL of sterile water	Laboratory	30.9*	Osmolarity	[22]
Astragalus	*Astragalus*	Iran	32	Acidic pH, hydrogen peroxide and high osmolarity	[18]
Chamomile	*Chamaemelum nobile*	Iran	44	Acidic pH, hydrogen peroxide and high osmolarity	[18]
Citrus	*Citrus*	Iran	42	Acidic pH, hydrogen peroxide and high osmolarity	[18]
Comvita 18+	*Leptospermum*	New Zealand	28.9*	Methylglyoxal	[22]
Heather	*Erica*	Portugal	>43.5*	Phenolic compounds. Potential chemical markers: ellagic acid and myricetin-30-methylether	[23]
Jarrah	*Eucalyptus marginata*	Australia	29*	Hydrogen peroxide and volatile and phenolic compounds: isophorone and Nonanoic acid	[21]
Jarrah	*Eucalyptus marginata*	Australia	13.4*	Hydrogen peroxide	[22]
Jellybush	*Leptospermum polygalifolium*	Australia	29*	Volatile and phenolic compounds: linalool and nonanal	[21]
Jujube	*Zizyphus spina-christi*	Saudi Arabia	29*	Interference with cell membrane integrity, affects exopolysaccharide composition of biofilms	[24]
Kelulut	Malaysian stingless bee	Malaysia	10	-	[19]
M. beecheii	Multifloral, *Melipona beecheii* bee	Mexico	35	Flavonoids cause loss of polarization of growth, cellular lysis and cell walls damage of C. albicans.	[20]
Mad Honey (>2 mg/mL)	*Rhododendron*	Turkey	0.1*	Phenolic compounds: linalool, phenethyl alcohol and benzyl alcohol	[25]
Manuka	*Leptospermum scoparium*	New Zealand	29*	Acetanisole and Methyl 3,5-dimethoxy benzoate	[21]

	Medihoney Barrier	Blend of *Leptospermum* and hydrogen peroxide honeys	-	27.7*	Methylglyoxal and hydrogen peroxide	[22]
	Multiflora	-	Iran	35	Acidic pH, hydrogen peroxide and high osmolarity	[18]
	Surgihoney RO™- low potency	-	-	1.2*	Hydrogen peroxide	[26]
	Surgihoney RO™- high potency	-	-	0.3*	Hydrogen peroxide	[26]
	Tea Tree	*Leptospermum lanigerum & Leptospermum scoparium*	Australia	29*	Hydrogen Peroxide and major volatile compounds: Acetanisole, and Methyl 3,5-dimethoxy benzoate	[21]
	Thymus	*Thymus vulgaris*	Iran	26	High phenolic content: caravacrol, thymol and gamma terpinene	[18]
	Tualang	Multifloral by *Apis dorsata* bees	Malaysia	25	-	[19]
	Zizyphus	*Zizyphus*	Iran	48	Acidic pH, hydrogen peroxide and high osmolarity	[18]
Candida dubliniensis	Alfalfa	*Alfalfa*	Iran	15	High phenolic content: tannins, pectin substances, saponines, amines, triterpene glycosides, purines base, phytoestrogens, flavones, isoflavonoids	[18]
	Artificial honey	-	-	24.9*	Osmolarity	[22]
	Astragalus	*Astragalus*	Iran	25	Acidic pH, hydrogen peroxide and high osmolarity	[18]
	Chamaemelum	*Chamaemelum nobile*	Iran	40	Acidic pH, hydrogen peroxide and high osmolarity	[18]
	Citrus	*Citrus*	Iran	35	Acidic pH, hydrogen peroxide and high osmolarity	[18]
	Comvita 18+	*Leptospermum*	New Zealand	24.2*	Methylglyoxal	[22]
	Jarrah	*Eucalyptus marginata*	Australia	11.2*	Hydrogen peroxide	[22]

	Medihoney Barrier	Blend of *Leptospermum* and hydrogen peroxide honeys	-	25.1*	Methylglyoxal and hydrogen peroxide	[22]
	Multiflora	Multifloral	Iran	30	Acidic pH, hydrogen peroxide and high osmolarity	[18]
	Thymus	*Thymus vulgaris*	Iran	20	High phenolic content: caravacrol, thymol and gamma terpinene	[18]
	Zizyphus	*Zizyphus*	Iran	45	Acidic pH, hydrogen peroxide and high osmolarity	[18]
Candida glabrata	Alfalfa	*Alfalfa*	Iran	23	High phenolic content: tannins, pectin substances, saponines, amines, triterpene glycosides, purines base, phytoestrogens, flavones, isoflavonoids	[18]
	Artificial honey	7.5 g sucrose, 37.5 g maltose, 167.5 g glucose, and 202.5 g fructose in 85 mL of sterile water	Laboratory	32.4*	Osmolarity	[22]
	Astragalus	*Astragalus*	Iran	30.5	Acidic pH, hydrogen peroxide and high osmolarity	[18]
	Chamaemelum	*Chamaemelum nobile*	Iran	47	Acidic pH, hydrogen peroxide and high osmolarity	[18]
	Citrus	*Citrus*	Iran	40	Acidic pH, hydrogen peroxide and high osmolarity	[18]
	Comvita 18+	*Leptospermum*	New Zealand	30.9*	Methylglyoxal	[22]
	Jarrah	*Eucalyptus marginata*	Australia	21.7*	Hydrogen peroxide	[22]
	Medihoney Barrier	Blend of *Leptospermum* and hydrogen peroxide honeys	-	31.2*	Methylglyoxal and hydrogen peroxide	[22]
	Multiflora	Multifloral	Iran	28.5	Acidic pH, hydrogen peroxide and high osmolarity	[18]
	Thymus	*Thymus vulgaris*	Iran	26	High phenolic content: caravacrol, thymol and gamma terpinene	[18]

	Zizyphus	*Zizyphus*	Iran	45	Acidic pH, hydrogen peroxide and high osmolarity	[18]
Candida kefyr	Alfalfa	*Alfalfa*	Iran	30	High phenolic content: tannins, pectin substances, saponines, amines, triterpene glycosides, purines base, phytoestrogens, flavones, isoflavonoids	[18]
	Astragalus	*Astragalus*	Iran	35	Acidic pH, hydrogen peroxide and high osmolarity	[18]
	Chamaemelum	*Chamaemelum nobile*	Iran	55	Acidic pH, hydrogen peroxide and high osmolarity	[18]
	Citrus	*Citrus*	Iran	55	Acidic pH, hydrogen peroxide and high osmolarity	[18]
	Multiflora	Multifloral	Iran	40	Acidic pH, hydrogen peroxide and high osmolarity	[18]
	Thymus	*Thymus vulgaris*	Iran	35	High phenolic content: caravacrol, thymol and gamma terpinene	[18]
	Zizyphus	*Zizyphus*	Iran	55	Acidic pH, hydrogen peroxide and high osmolarity	[18]
Candida krusei	Alfalfa	*Alfalfa*	Iran	23.5	High phenolic content: tannins, pectin substances, saponines, amines, triterpene glycosides, purines base, phytoestrogens, flavones, isoflavonoids	[18]
	Astragalus	*Astragalus*	Iran	32	Acidic pH, hydrogen peroxide and high osmolarity	[18]
	Chamaemelum	*Chamaemelum nobile*	Iran	49.5	Acidic pH, hydrogen peroxide and high osmolarity	[18]
	Citrus	*Citrus*	Iran	42	Acidic pH, hydrogen peroxide and high osmolarity	[18]
	Heather	*Erica*	Portugal	43.5*	Phenolic compounds. Potential chemical markers: ellagic acid and myricetin-30-methylether	[23]
	Multiflora	Multifloral	Iran	35	Acidic pH, hydrogen peroxide and high osmolarity	[18]
	Thymus	*Thymus vulgaris*	Iran	23.5	High phenolic content: caravacrol, thymol and gamma terpinene	[18]
	Zizyphus	*Zizyphus*	Iran	50	Acidic pH, hydrogen peroxide and high osmolarity	[18]
Candida parapsilosis	Alfalfa	*Alfalfa*	Iran	25	High phenolic content: tannins, pectin substances, saponines, amines, triterpene glycosides, purines base, phytoestrogens, flavones, isoflavonoids	[18]
	Astragalus	*Astragalus*	Iran	30	Acidic pH, hydrogen peroxide and high osmolarity	[18]
	Chamaemelum	*Chamaemelum nobile*	Iran	45	Acidic pH, hydrogen peroxide and high osmolarity	[18]
	Citrus	*Citrus*	Iran	40	Acidic pH, hydrogen peroxide and high osmolarity	[18]

	Multiflora	Multifloral	Iran	35	Acidic pH, hydrogen peroxide and high osmolarity	[18]
	Thymus	*Thymus vulgaris*	Iran	25	High phenolic content: caravacrol, thymol and gamma terpinene	[18]
	Zizyphus	*Zizyphus*	Iran	50	Acidic pH, hydrogen peroxide and high osmolarity	[18]
Candida neoformans	Heather	*Erica*	Portugal	>43.5*	Phenolic compounds. Potential chemical markers: ellagic acid and myricetin-30-methylether	[23]
Candida tropicalis	Alfalfa	*Alfalfa*	Iran	25	High phenolic content: tannins, pectin substances, saponines, amines, triterpene glycosides, purines base, phytoestrogens, flavones, isoflavonoids	[18]
	Astragalus	*Astragalus*	Iran	30	Acidic pH, hydrogen peroxide and high osmolarity	[18]
	Chamaemelum	*Chamaemelum nobile*	Iran	46	Acidic pH, hydrogen peroxide and high osmolarity	[18]
	Citrus	*Citrus*	Iran	43	Acidic pH, hydrogen peroxide and high osmolarity	[18]
	Multiflora	Multifloral	Iran	35	Acidic pH, hydrogen peroxide and high osmolarity	[18]
	Thymus	*Thymus vulgaris*	Iran	26.5	High phenolic content: caravacrol, thymol and gamma terpinene	[18]
	Zizyphus	*Zizyphus*	Iran	46	Acidic pH, hydrogen peroxide and high osmolarity	[18]
Trichophyton mentagrophytes	Agastache	*Agastache Rugosa*	Australia	29*	Phenolic compounds: Phenol, 2,4-bis(1,1-dimethylethyl), estragole and nonanoic acid and ethyl ester	[21]
	Tea Tree	*Leptospermum lanigerum & Leptospermum scoparium*	Australia	29*	Hydrogen Peroxide and major volatile compounds: Acetanisole, and Methyl 3,5-dimethoxy benzoate	[21]
Trichophyton rubrum	Agastache	*Agastache Rugosa*	Australia	29*	Phenolic compounds: Phenol, 2,4-bis(1,1-dimethylethyl), estragole and nonanoic acid and ethyl ester	[21]
	Alfalfa	*Alfalfa*	Iran	20	High phenolic content: tannins, pectin substances, saponines, amines, triterpene glycosides, purines base, phytoestrogens, flavones, isoflavonoids	[18]
	Astragalus	*Astragalus*	Iran	31	Acidic pH, hydrogen peroxide and high osmolarity	[18]

Chamaemelum	*Chamaemelum nobile*	Iran	43	Acidic pH, hydrogen peroxide and high osmolarity	[18]
Citrus	*Citrus*	Iran	39	Acidic pH, hydrogen peroxide and high osmolarity	[18]
Manuka	*Leptospermum scoparium*	New Zealand	29*	Acetanisole, and Methyl 3,5-dimethoxy benzoate	[18]
Multiflora	Multifloral	Iran	25	Acidic pH, hydrogen peroxide and high osmolarity	[18]
Thymus	*Thymus vulgaris*	Iran	22	High phenolic content: caravacrol, thymol and gamma terpinene	[18]
Zizyphus	*Zizyphus*	Iran	44	Acidic pH, hydrogen peroxide and high osmolarity	[18]

* MIC values converted from %w/v to %v/v using the honey density of 1.38g/mL (lowest reported density), to report the highest possible MIC.

References

1. Comvita. UMF™ Manuka Honey. Available online: <https://www.comvita.co.uk/category/umf-manuka-honey/range00001> (accessed on 28 August 2020).
2. Egmonthoney. Products. Available online: <https://egmonthoney.co.uk/collections/all> (accessed on 28 August 2020).
3. Manuka Doctor. Manuka Honey. Available online: <https://www.manukadoctor.co.uk/collections/manuka-honey> (accessed on 28 August 2020).
4. Savant Distribution. MGO Manuka Honey. Available online: <https://www.manukahealthuk.com/shop/mgo-manuka-honey.html> (accessed on 28 August 2020).
5. Manuka Pharm. Manuka Honey. Available online: <https://www.manukapharm.co.uk/collections/manuka-honey> (accessed on 28 August 2020).
6. Melora. MELORA MĀNUKA HONEY. Available online: <https://www.melora.co.uk/collections/manuka-honey-food> (accessed on 28 August 2020).
7. Optima Health and Nutrition. PREMIUM SELECT MANUKA HONEY. Available online: <https://optimah.com/pages/pure-gold-premium-manuka-honey> (accessed on 28 August 2020).
8. Tan, H.T.; Rahman, R.A.; Gan, S.H.; Halim, A.S.; Hassan, S.A.; Sulaiman, S.A.; Kirnpal-Kaur, B.S. The antibacterial properties of Malaysian tualang honey against wound and enteric microorganisms in comparison to manuka honey. *BMC Complement. Altern. Med.* **2009**, *9*, 8, doi:10.1186/1472-6882-9-34.
9. Cooper, R.A.; Molan, P.C.; Harding, K.G. The sensitivity to honey of Gram-positive cocci of clinical significance isolated from wounds. *J. Appl. Microbiol.* **2002**, *93*, 857-863, doi:10.1046/j.1365-2672.2002.01761.x.
10. Grego, E.; Robino, P.; Tramuta, C.; Giusto, G.; Boi, M.; Colombo, R.; Serra, G.; Chiado-Cutin, S.; Gandini, M.; Nebbia, P. Evaluation of antimicrobial activity of Italian honey for wound healing application in veterinary medicine. *Schweiz. Arch. Tierheilkd.* **2016**, *158*, 521-527, doi:10.17236/sat00075.
11. Goslinski, M.; Nowak, D.; Klebukowska, L. Antioxidant properties and antimicrobial activity of manuka honey versus Polish honeys. *J. Food Sci. Technol.-Mysore* **2020**, *57*, 1269-1277, doi:10.1007/s13197-019-04159-w.
12. Anand, S.; Deighton, M.; Livanos, G.; Morrison, P.D.; Pang, E.C.K.; Mantri, N. Antimicrobial Activity of Agastache Honey and Characterization of Its Bioactive Compounds in Comparison With Important Commercial Honeys. *Front. Microbiol.* **2019**, *10*, 16, doi:10.3389/fmicb.2019.00263.
13. Grecka, K.; Kus, P.M.; Worobo, R.W.; Szweda, P. Study of the Anti-Staphylococcal Potential of Honeys Produced in Northern Poland. *Molecules* **2018**, *23*, 23, doi:10.3390/molecules23020260.
14. Anthimidou, E.; Mossialos, D. Antibacterial Activity of Greek and Cypriot Honeys Against Staphylococcus aureus and Pseudomonas aeruginosa in Comparison to Manuka Honey. *J. Med. Food* **2013**, *16*, 42-47, doi:10.1089/jmf.2012.0042.
15. Miorin, P.L.; Junior, N.C.L.; Custodio, A.R.; Bretz, W.A.; Marcucci, M.C. Antibacterial activity of honey and propolis from Apis mellifera and Tetragonisca angustula against Staphylococcus aureus. *J. Appl. Microbiol.* **2003**, *95*, 913-920, doi:10.1046/j.1365-2672.2003.02050.x.
16. Sherlock, O.; Dolan, A.; Athman, R.; Power, A.; Gethin, G.; Cowman, S.; Humphreys, H. Comparison of the antimicrobial activity of Ulmo honey from Chile and Manuka honey against methicillin-resistant Staphylococcus aureus, Escherichia coli and Pseudomonas aeruginosa. *BMC Complement. Altern. Med.* **2010**, *10*, 5, doi:10.1186/1472-6882-10-47.
17. Boukraa, L. Additive Activity of Royal Jelly and Honey Against Pseudomonas aeruginosa. *Altern. Med. Rev.* **2008**, *13*, 330-333.

18. Katiraee, F.; Mahmodi, R.; Mardani, K.; Babaei, E. ANTIFUNGAL ACTIVITY OF IRANIAN HONEYBEES AGAINST CANDIDA, ASPERGILLUS SPECIES AND TRICHOPHYTON RUBRUM. *J. Food Process Preserv.* **2014**, *38*, 2078-2082, doi:10.1111/jfpp.12187.
19. Hamid, Z.; Mohamad, I.; Harun, A.; Salim, R.; Sulaiman, S.A. Antifungal Effect of Three Local Malaysian Honeys on Selected Pathogenic Fungi of Otomycosis: An in vitro Evaluation. *J. Young Pharm.* **2018**, *10*, 414-417, doi:10.5530/jyp.2018.10.91.
20. Hau-Yama, N.E.; Magana-Ortiz, D.; Oliva, A.I.; Ortiz-Vazquez, E. Antifungal activity of honey from stingless bee Melipona beecheii against Candida albicans. *Journal of Apicultural Research* **2020**, *59*, 12-18, doi:10.1080/00218839.2019.1665247.
21. Anand, S.; Deighton, M.; Livanos, G.; Pang, E.C.K.; Mantri, N. Agastache honey has superior antifungal activity in comparison with important commercial honeys. *Sci Rep* **2019**, *9*, 14, doi:10.1038/s41598-019-54679-w.
22. Irish, J.; Carter, D.A.; Shokohi, T.; Blair, S.E. Honey has an antifungal effect against Candida species. *Med. Mycol.* **2006**, *44*, 289-291, doi:10.1080/13693780500417037.
23. Feas, X.; Estevinho, M.L. A Survey of the In Vitro Antifungal Activity of Heather (Erica Sp.) Organic Honey. *J. Med. Food* **2011**, *14*, 1284-1288, doi:10.1089/jmf.2010.0211.
24. Ansari, M.J.; Al-Ghamdi, A.; Usmani, S.; Al-Waili, N.S.; Sharma, D.; Nuru, A.; Al-Attal, Y. Effect of Jujube Honey on Candida albicans Growth and Biofilm Formation. *Arch. Med. Res.* **2013**, *44*, 352-360, doi:10.1016/j.arcmed.2013.06.003.
25. Erturk, O.; Karakas, F.P.; Pehlivan, D.; Nas, N. The Antibacterial and Antifungal Effects of Rhododendron Derived Mad Honey and Extracts of Four Rhododendron Species. *Turk. J. Biol.* **2009**, *33*, 151-158.
26. Dryden, M.; Lockyer, G.; Saeed, K.; Cooke, J. Engineered honey: In vitro antimicrobial activity of a novel topical wound care treatment. *J. Glob. Antimicrob. Resist.* **2014**, *2*, 168-172, doi:10.1016/j.jgar.2014.03.006.
